# Regulatory conservation of protein coding and microRNA genes in vertebrates: lessons from the opossum genome

**DOI:** 10.1186/gb-2007-8-5-r84

**Published:** 2007-05-16

**Authors:** Shaun Mahony, David L Corcoran, Eleanor Feingold, Panayiotis V Benos

**Affiliations:** 1 Department of Computational Biology, School of Medicine, University of Pittsburgh, Fifth Avenue, Pittsburgh, PA 15260, USA; 2 Department of Human Genetics, Graduate School of Public Health, University of Pittsburgh, DeSoto Street, Pittsburgh, PA 15261, USA; 3 Department of Biostatistics, Graduate School of Public Health, University of Pittsburgh, DeSoto Street, Pittsburgh, PA 15261, USA; 4 University of Pittsburgh Cancer Institute, School of Medicine, University of Pittsburgh, Centre Avenue, Pittsburgh, PA 15232, USA

## Abstract

**Background:**

Being the first noneutherian mammal sequenced, *Monodelphis domestica *(opossum) offers great potential for enhancing our understanding of the evolutionary processes that take place in mammals. This study focuses on the evolutionary relationships between conservation of noncoding sequences, *cis*-regulatory elements, and biologic functions of regulated genes in opossum and eight vertebrate species.

**Results:**

Analysis of 145 intergenic microRNA and all protein coding genes revealed that the upstream sequences of the former are up to twice as conserved as the latter among mammals, except in the first 500 base pairs, where the conservation is similar. Comparison of promoter conservation in 513 protein coding genes and related transcription factor binding sites (TFBSs) showed that 41% of the known human TFBSs are located in the 6.7% of promoter regions that are conserved between human and opossum. Some core biologic processes exhibited significantly fewer conserved TFBSs in human-opossum comparisons, suggesting greater functional divergence. A new measure of efficiency in multigenome phylogenetic footprinting (base regulatory potential rate [BRPR]) shows that including human-opossum conservation increases specificity in finding human TFBSs.

**Conclusion:**

Opossum facilitates better estimation of promoter conservation and TFBS turnover among mammals. The fact that substantial TFBS numbers are located in a small proportion of the human-opossum conserved sequences emphasizes the importance of marsupial genomes for phylogenetic footprinting-based motif discovery strategies. The BRPR measure is expected to help select genome combinations for optimal performance of these algorithms. Finally, although the etiology of the microRNA upstream increased conservation remains unknown, it is expected to have strong implications for our understanding of regulation of their expression.

## Abstract 2

A study of conservation of non-coding sequences, *cis*-regulatory elements and biological functions of regulated genes in opossum and other vertebrates enables better estimation of promoter conservation and transcription factor binding site turnover among mammals

## Background

One of the prime motivating factors driving the sequencing of vertebrate genomes is the expectation that the role played by the functional regions of the human genome may be discerned by finding molecular level commonalities with and differences from other animals. This is especially true of the newly sequenced opossum (*Monodelphis domestica*), which is the first completed marsupial genome. Being the first noneutherian mammal sequenced, the opossum helps to clarify which sequence changes occurred before and after the divergence of mammalian ancestors from other vertebrates [1], and has already provided new insight into the evolution of mammalian major histocompatibility complex genes [2]. It is also hoped that the opossum genome may yield insights into how gene regulation has evolved in vertebrates.

In protein coding genes, gene regulation is primarily controlled by short DNA sequences in the vicinity of the gene's transcription start sites (TSSs), which are targets for transcription factor proteins. A high degree of evolutionary conservation of these promoter regions can be attributed to functional *cis*-regulatory elements. The increased conservation in the biologically more important parts of the promoter region has been explored by various phylogenetic footprinting algorithms, such as PhyloGibbs [3], ConSite [4], rVista [5], and FOOTER [6], to improve the prediction of transcription factor binding sites (TFBSs) in vertebrate genomes. Phylogenetic footprinting is a comparative genomics approach that exploits cross-species sequence conservation in order to predict regulatory genomic elements. In the absence of evolutionary information, TFBSs can be evaluated in terms of sequence similarity scans against frequency matrices derived from alignments of known binding sites for a given transcription factor [7]. However, the typical short length of TFBSs (5 to 20 base pairs [bp]) and their inherent level of sequence degeneracy makes them notoriously difficult to predict with any degree of specificity using similarity searches alone [8]. Phylogenetic footprinting provides a way to reduce the sequence search space to regions that are conserved (and therefore more likely to contain functional elements), thereby improving the specificity of TFBS prediction.

In order to improve the performance of phylogenetic footprinting algorithms, the evolutionary aspects of the promoter regions and the TFBSs residing in them must be investigated. Evolutionary distance is an important factor in the effectiveness of phylogenetic footprinting techniques. For example, the divergence between chimpanzee and human is generally insufficient to reduce the sequence search space in any meaningful way; conversely, the divergence between *Drosophila *and human can be too large for any regulatory sequence conservation to be detected. Recently, the maximum sensitivity of phylogenetic footprinting techniques has been measured via estimations of the rate of TFBS 'turnover' between human and rodent genomes [9-13]. We consider that a TFBS has undergone turnover if the sequence in which it resides is not conserved between the species compared. High or low TFBS turnover rates do not necessarily coincide with the rate of changes in the regulatory mechanism (for instance, replacement TFBSs can arise by chance elsewhere in the promoter region or functional TFBSs may still be present in nonconserved regions). Turnover, however, corresponds to the minimum false-negative rate for detection of TFBSs via phylogenetic footprinting, and thus it serves as a critical bound on the success of such algorithms. Human-rodent TFBS turnover has been estimated at between 28% and 40% [9-13], suggesting that TFBSs are among the most malleable functional elements in the genomic landscape. However, although rodents and primates diverged relatively recently (approximately 90 million years ago [14]), the shorter generational time of rodents has placed a large degree of dissimilarity between the two clades, as is evident in the human-dog comparisons [15]. Therefore, TFBS turnover rates will have to be estimated in other mammals before a clearer picture of the selective pressure on mammalian TFBSs can emerge.

Another major mechanism for control of gene expression is provided by microRNA (miRNA) genes. miRNAs are small (22 to 61 bp long), noncoding RNAs that downregulate their target genes via base complementarity to their mRNA molecules [16,17]. Each miRNA can target multiple genes and each gene can be targeted by multiple miRNAs [18-21]. In vertebrates, their expression is tissue specific [22] and has been shown to play an important role during development [23-25]. Although some miRNAs are found in the introns of coding genes and therefore are probably regulated by the promoters of the genes in which they reside [26], others are located in the intergenic parts of the genome. Little is known about the transcriptional regulation of these intergenic miRNAs, although RNA polymerase II appears to be involved in the process [27]. This suggests that they may have active promoter regions that contain *cis*-regulatory elements, similar to coding genes. The following question then arises; how does the conservation in the upstream regions of the intergenic miRNA genes compare with that of the protein coding genes? In this respect, opossum and the other vertebrate species provide a broad range of evolutionary distances in which this issue may be addressed.

In this report we present our findings regarding promoter conservation of all protein coding genes and upstream sequence conservation of intergenic miRNA genes in eight vertebrate genomes as compared with human. To our knowledge, this is the first time that such a comprehensive study has been conducted on potential regulatory regions of both protein coding and miRNA genes in vertebrates. Also, because the opossum genome is placed at an evolutionary midpoint relative to eutherian mammals and nonmammalian vertebrates, using it as an outgroup to the existing eutherian genomes allows for the estimation of the mammalian TFBS turnover rate. Furthermore, the opossum genome provides an opportunity to assess which transcriptional signals and regulatory mechanisms are shared between all mammals. For these reasons, the conservation rates of the promoters of 513 human genes are also analyzed in relation to the turnover of the 1,162 TFBSs they contain. Relationships between conservation of sites and identity of the corresponding transcription factors and their Gene Ontology (GO) [28] categories are also investigated. Finally, we computationally re-evaluate the potential of phylogenetic footprinting in the light of the opossum genome and other recently sequenced vertebrates. A new statistical measure, the base regulatory potential rate (BRPR), is introduced to assess the efficiency of both pair-wise and multiple species comparisons in phylogenetic footprinting strategies.

## Results and discussion

### Distribution of conserved blocks in the upstream regions of protein coding and intergenic miRNA genes

**Figure 1:**
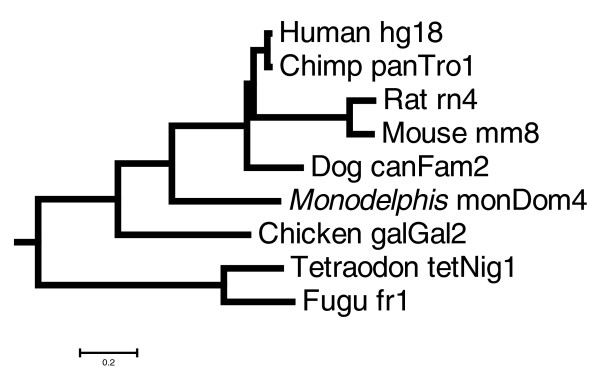
Phylogenetic tree of the species examined in this study. This phylogenetic tree is based on the University of California, Santa Cruz (UCSC) multiple alignments. The tree was generated using phyloGif [72].

Conservation of the 5 kilobases (kb) upstream regions of all RefSeq protein coding genes as well as the known intergenic miRNA genes was calculated using the sliding window approach, as we describe in Materials and methods (below). We chose to focus solely on intergenic miRNAs because intronic miRNAs have been shown to be co-transcribed with their corresponding protein coding genes [26]. Because little is known about the transcriptional regulation of non-intronic miRNA genes, we cannot assess the possible TFBS turnover. We can, however, assess whether the miRNA upstream regions evolve at the same, slower, or faster rate than those of the protein coding genes, and whether their conservation pattern across the upstream region indicates parts of potential biologic importance. The phylogenetic tree of the species examined in this paper is plotted in Figure 1.

Table 1 presents the number of orthologous genes in each species (derived from the MULTIZ University of California, Santa Cruz [UCSC] synteny-based alignments), the average block coverage of their upstream regions, and the average percentage identity within these conserved blocks. For the calculation of the average percentage identity, the conservation percentage of each block is multiplied by the total length of the block. In other words, the average block conservation corresponds to the number of bases that are identical in all conserved blocks of one promoter over the total length of the blocks in this promoter. The human genes were used as reference for all pair-wise comparisons. Surprisingly, we found that, with the exception of teleosts and chimp, the conservation in the upstream regions of the miRNA genes is 34% to 60% higher on average than that in the protein coding genes. This is independent of the average block identity, which remains practically the same between the two types of genes in these comparisons (Table 1). In all nonprimate mammals the average block coverage in the miRNA upstream sequences is significantly higher than that in the promoters of the protein coding genes (Wilcoxon rank-sum test: *P *= 6 × 10^-4 ^for opossum and *P *= 10^-14 ^to 10^-16 ^for rodents and dog).

**Table 1:** Conservation in the 5 kilobases upstream sequences in all protein coding and intergenic miRNA genes

Human versus	Protein coding genes			Intergenic miRNA genes			Relative conservation
			
	Number of orthologous	Block coverage	Average block identity	Number of orthologous	Block coverage	Average block identity	
Chimp	23,643	93.03%	98.15%	144	93.46%	98.51%	0.46%
Mouse*	22,790	23.30%*	73.53%	142	36.17%*	74.72%	55.24%
Rat*	22,161	22.46%*	73.49%	140	34.95%*	74.68%	55.61%
Dog*	23,276	44.36%*	75.58%	145	61.72%*	76.96%	39.13%
Opossum*	17,334	7.28%*	74.90%	104	11.65%*	76.08%	60.03%
Chicken	8,087	4.55%	74.87%	54	6.08%	76.80%	33.63%
Fugu	6,257	4.13%	72.17%	47	2.73%	73.65%	-33.90%
Tetraodon	7,821	3.43%	72.10%	60	2.31%	73.40%	-32.65%

This table lists the number of genes orthologous to human genes in each of the genomes tested, the percentage of upstream sequence conservation (in >65% block identity), and the weighted average within block identity. Relative conservation (in terms of block coverage) is also listed for the microRNA (miRNA) versus protein coding genes. *Species for which the block coverage of miRNA gene upstream regions is statistically significantly higher than that of the promoters of the protein coding genes.

**Figure 2:**
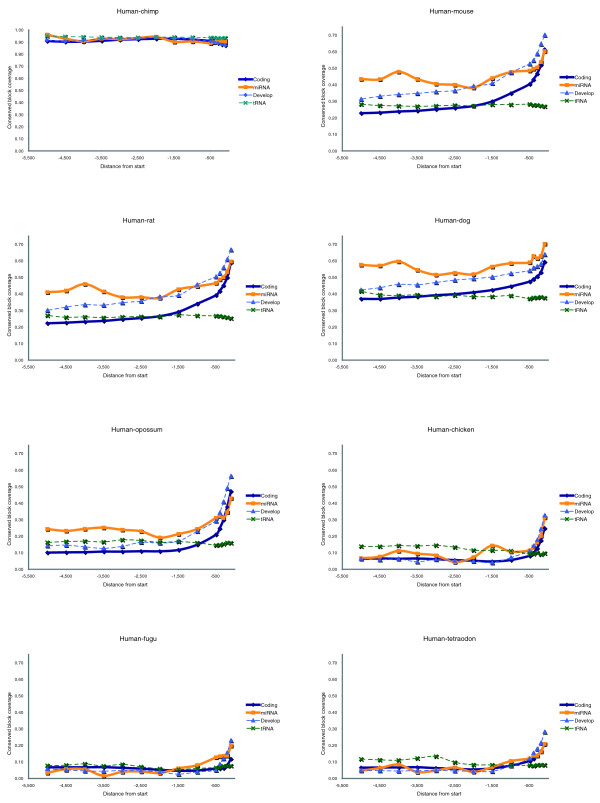
Upstream sequence conservation of protein coding versus miRNA genes. Comparison of 5-kilobase upstream sequence conservation between human and various organisms, relative to the transcription start site (TSS; protein-coding, solid blue line) and gene start (intergenic microRNA [miRNA] genes, orange line). The conservation of developmental genes (light blue dotted line) and tRNA genes (green dotted line) are also plotted for comparison purposes. For the plot 100 base pair (bp) intervals were used for the first 500 bp and 500 bp intervals thereafter.

In order to investigate this surprising finding further, we plotted the sequence conservation as a function of the distance from the start of the corresponding genes (Figure 2). We found that in the first 500 bp the sequence conservation of the miRNA genes is almost identical to that of the promoters of the protein coding genes (*R *values > 0.9 and usually much higher; regression *t*-test: *P *< 10^-19^). In protein coding genes this is typically the region with the highest concentration of the known *cis*-regulatory elements. From all known human and mouse TFBSs in TRANSFAC [29], 69.1% and 65.1%, respectively, are annotated as being located in the proximal 500 bp region (data not shown). Interestingly, Lee and coworkers [27] showed that this region is sufficient to drive expression of the miR 23a~27a~24-2 intergenic miRNA gene cluster by RNA polymerase II. Could this be a coincidence? We tested this by analyzing the upstream sequence conservation of the tRNA genes in the human genome (see Materials and methods, below). It has been long established that the *cis*-regulatory elements of the tRNA genes are located downstream of their transcription start [30]. We found that the sequence conservation for the tRNA genes was constant throughout their 5 kb upstream regions (Figure 2; green dashed line).

The conservation rates in both protein coding and miRNA genes decline after the first 500 bp and become almost constant. The difference between these two types of genes is that, in the case of miRNAs, the constant conservation rate is up to twofold higher than that in the protein coding genes for rodents, dog, opossum, and chicken. We found this difference to be statistically significant (Additional data file 1 [Supplementary Figure 2]). Similarly high conservation rates are observed in chimp for both types of genes, probably reflecting the generally high conservation rate throughout the genome. By contrast, similarly low conservation rates are observed for the fugu fish and tetraodon. We note, however, that the higher conservation rates are statistically significant only in the (nonprimate) mammals, including opossum (Additional data file 1).

It is not clear whether this increased upstream sequence conservation is a general biologic feature of the miRNA upstream regions or is an artifact of the methods used to discover miRNA genes. It is possible, for example, that the known intergenic miRNAs happen to fall in more conserved regions of the genome. This may be related to the way in which the miRNAs were originally identified (through high similarity to known miRNAs). However, it is also possible that because miRNAs are involved in highly regulated vital cell or organismal processes such as development [23-25], there is a much greater selective pressure on their regulatory regions. We investigate this further by comparing the upstream sequence conservation in the miRNA genes with that of genes identified as developmental according to GO classification (Figure 2; light blue dashed line). We find that the upstream conservation of the developmental genes in all mammals is uniformly higher than the overall average and similar to the conservation of the miRNA genes, especially in the first 2,000 bp. This is true for all species examined, although in the nonmammalian vertebrates the overall upstream sequence conservation for all types of genes is similarly low (10% or lower after the first 500 bp; Figure 2). The fact that miRNA genes have been implicated in the regulation of various developmental processes [31] may partly explain the similar conservation rates in their upstream regions and the promoters of the developmental genes, also indicating that analogous mechanisms and *cis*-elements may regulate the expression of the corresponding genes. The fact that opossum sequences also exhibit similar conservation patterns, as do the sequences of eutherian species, indicates that mammalian specific evolutionary constraints are in place.

In summary, the above observations are consistent with the idea that miRNAs are regulated by similar mechanisms as protein coding genes, which was also shown to be true in the few cases studied thus far [27,32]. As more miRNA genes are identified, the issue of their transcriptional mechanism will warrant further investigation.

In all of the above pair-wise comparisons, except human-chimp, the average block identity is about the same (72% to 77%; Table 1), regardless of the evolutionary distance or the type of gene (protein coding or miRNA). Because the block conservation threshold was 65%, this equivalency indicates that a reduction in the number of conserved blocks rather than a uniform decrease in similarity is responsible for the observed conservation rates. Such a pattern of evolution is expected if the *cis*-regulatory sites are organized in clusters located in these upstream regions. Such clusters might contain regulatory elements specific to, for instance, primates only, eutherians only, and so on.

### Evolutionary turnover of transcription factor binding sites in vertebrates

We now turn to the relationship between promoter conservation of the protein coding genes and the turnover of the *cis*-regulatory elements located in them. Table 2 presents the percentage of known human TFBSs that reside in conserved blocks for each pair of genomes tested. The number of such detectable TFBSs in each species differs depending on the number of orthologous genes identified in that species. We note that our analysis focuses on the TFBSs that are located immediately upstream of the protein coding genes (up to 5 kb). This bias is imposed by the available data. It will be interesting to see how our results compare with the evolution of DNA regulatory regions in other parts of the genome.

**Table 2:** Promoter and site conservation between human and eight vertebrate species

Human versus	Promoters			Sites			BRPR
			
	Number of orthologous genes	Block coverage	Block nucleotide identity	Number of detectable sites	% detected	Site nucleotide identity	
Chimp	512	94.06%	98.27%	1,157	94.81%	98.74%	1.009
Mouse	506	24.20%	73.39%	1,146	72.34%	82.91%	2.887
Rat	496	23.09%	73.21%	1,129	67.14%	83.00%	2.757
Dog	507	46.05%	75.37%	1,151	73.59%	84.77%	1.535
Opossum	389	6.72%	74.63%	912	41.23%	83.93%	5.647
Chicken	189	3.21%	74.43%	451	21.73%	85.06%	6.184
Fugu	127	3.25%	72.87%	286	11.89%	83.98%	3.331
Tetraodon	166	2.50%	73.09%	363	12.12%	80.95%	4.227

Analysis of 1,162 known human transcription factor binding sites (TFBSs) associated with the promoters of 513 human genes between human and eight vertebrate species. The number of genes orthologous to human genes in each species, their conservation block coverage, and their average block identity are presented; also, the number of TFBSs associated with these orthologous genes in each species, the percentage of sites located in conserved regions between species, and the average nucleotide identity within TFBSs are reported. The base regulatory potential rate (BRPR) statistic is calculated from these data for each pair of genomes (see text). Block coverage is the percentage of the upstream region that is covered by conserved blocks (>50 base pairs with >65% identity); the block nucleotide identity is the percentage of nucleotides in all conserved blocks that are identical to the human sequence; and site nucleotide identity the percentage nucleotides in all detected TFBSs that are identical to the human sequence.

Although we confirm previously estimated rate of human-mouse TFBS turnover [9-13], it is particularly interesting that 27% or more of the known human TFBSs are not located in blocks conserved in mammals more distant than rodents (Table 2). This does not necessarily mean that the mechanisms of gene regulation have changed accordingly. Functionally equivalent TFBSs are not always located in conserved blocks, as demonstrated in a recent comparison of gene regulation in human and zebrafish RET genes [33]. Similarly, individual TFBSs that are not conserved between two species may have been functionally replaced by other sites for the same transcription factor in one of the species [34]. The finding that only about 41% of TFBSs are located in conserved human-opossum blocks is nevertheless surprising, because it points to the relative ease with which individual mammalian TFBSs may be deleted, replaced, or added.

**Figure 3:**
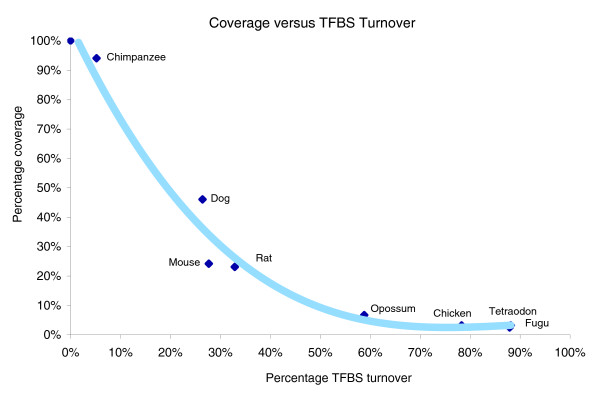
Conserved block coverage of the 5 kilobases upstream regions versus TFBS turnover rates. A third-order polynomial trendline is fitted for illustration. TFBS, transcription factor binding site.

As expected, TFBS turnover increases with decreasing percentage conservation coverage of the upstream regions. Figure 3 shows that opossum has low block conservation similar to that in the nonmammal vertebrate species, but it retains almost twice as many sites as chicken, which is the evolutionarily closest nonmammal. This gives a first qualitative assessment for the potential importance of the opossum genome for identification of TFBSs in phylogenetic footprinting approaches. In general, outside mammalian genomes, the percentage of the detected TFBSs is reduced with increasing evolutionary distance, although the percentage 5 kb upstream coverage remains constant.

Table 2 also presents the average identity within the conserved TFBSs. With the exception of human-chimp comparisons, the average identity within sites is substantially higher than the average identity in the conserved blocks and relatively constant in all genome comparisons. We found no linear correlation between the block coverage rate and the average block identity in these comparisons (*R *= 0.48). This finding supports the idea that individual TFBSs are under greater selective pressure than are the wider conserved blocks in mammalian genomes (Wilcoxon test: *P *= 0.01).

Finally, Table 2 presents the BRPR values for each pair of genomes (see Materials and methods, below). BRPR is the likelihood ratio of the posterior probability of a base being regulatory (part of a regulatory site), given that it is in a conserved region, over the *a priori *probability of being regulatory. In other words, BRPR shows how much we can improve our belief that a base (or a conserved region) is regulatory if we only focus on the conserved blocks between two or more species. One of the most surprising aspects of this study is that, on average, a relatively large percentage of TFBSs (41%) is located in only the 6.72% of the 5 kb promoter regions that are conserved between human and opossum. This gives human-opossum comparisons the second highest BRPR value among the tested pair-wise comparisons, and makes the use of opossum almost twice as effective for finding regulatory elements as the more typically used human-mouse alignments (BRPR 5.647 versus 2.887, respectively). Another interesting finding is that, because of the extensive conservation between human and dog genomes, the human-dog comparisons are not as effective as human-mouse for phylogeny-based motif discovery (Table 2). The maximum BRPR value occurs for human-chicken comparisons (BRPR 6.184). However, this value is very close to the opossum BRPR value and, given that only 22% of known TFBSs can be detected as conserved between human and chicken (as opposed to 41% in human-opossum), we suggest that human-opossum comparisons are more effective overall than human-chicken comparisons.

Phylogenetic footprinting becomes less effective in human-fugu and human-tetraodon comparisons (Table 2). The Afrotherian (elephant and tenrec) or Xenarthran (armadillo) genomes that are currently undergoing low-coverage sequencing, as well as the genomes of more distant vertebrates, do not appear to offer any improvement in pair-wise phylogenetic footprinting effectiveness (all are less effective than using the mouse genome; unpublished data). However, they may offer improvement in specificity in multispecies regulatory conservation scans.

### Phylogenetic footprinting with multispecies alignments

Thus far, the TFBS turnover rates and BRPR values were used in pair-wise comparisons in order to assess the relative effectiveness of discovering TFBSs via evolutionary conservation. Given the availability of multiple vertebrate genomes, it is naturally expected that combining conservation information from multiple sources will increase the accuracy of phylogenetic footprinting. The following question then arises; which genome combinations offer greater specificity? To address this, we evaluate all possible combinations of tested genomes (256 combinations). In the following, *P*(*C*) and *P*(*C|R*) are the prior and posterior probability, respectively, that a base is conserved, given that the base is part of a regulatory site. For consistency, both *P*(*C*) and *P*(*C|R*) are calculated over all known human sites in our dataset (1,162 sites) in all examined human upstream bases (513 genes × 5,000 bp = 2.565 megabases), regardless of the species we compare.

Table 3 shows the BRPR values for all comparisons between human and two other species. Interestingly, the highest *BRPR *value in three species comparisons is achieved when human sequences are compared with both opossum and chicken (BRPR 7.26). However, only 92 of the 1,162 known human TFBSs (7.9%) may be found via this strategy. Table 3 also shows that requiring a base to be conserved with both mouse and opossum is more effective than using either genome alone, and 31.7% of known human TFBSs may be detected in this way. The results of all tests (256 combinations) are provided in Additional data file 1. The combination with the overall highest BRPR value was human with chimp, mouse, opossum, and chicken (BRPR 7.628). We note that this maximum BRPR score places a cap on the possible value of *P*(*R*). In the unlikely event that all human-chimp-mouse-opossum-chicken conserved bases are part of TFBSs (that is, assuming *P*(*R|C*) = 1), then the maximum value of *P*(*R*) from Equation 1 (see materials and methods, below) is (7.628)^-1^. If we extrapolate, then we find that a maximum of 655 bp may be regulatory in the average human 5 kb upstream region. Taking the average size of a TFBS in the JASPAR database [35] of high-quality binding sites (10.658 bp) suggests that no more than 61.5 nonoverlapping TFBSs are present in the average 5 kb upstream region. This maximum value is in agreement with previous reports that estimate this number to be between 10 and 50 sites, depending on the promoter [36,37]. The addition of six more (as yet unpublished) vertebrate species in this analysis did not yield a combination of genomes with a higher BRPR than the human-chimp-mouse-opossum-chicken combination (data not shown).

**Table 3:** Three-way comparisons between human and two other vertebrate species

Human versus	Chimp	Mouse	Rat	Dog	Opossum	Chicken	Fugu	Tetraodon
Chimp		67.90%	62.48%	70.65%	31.67%	8.26%	2.75%	3.53%
Mouse	2.896		61.10%	59.29%	31.67%	8.35%	2.93%	3.79%
Rat	2.794	3.277		54.22%	29.43%	8.00%	2.58%	3.44%
Dog	1.561	3.070	2.940		27.54%	6.88%	2.93%	3.79%
Opossum	5.845	6.430	6.247	5.565		7.92%	2.75%	3.70%
Chicken	5.864	6.939	6.875	5.891	7.262*		1.29%	1.20%
Fugu	2.625	3.409	3.207	3.457	3.604	2.891		2.67%
Tetraodon	3.195	4.103	3.951	4.165	4.620	2.775	3.468	

Base regulatory potential rate (BRPR) for bases conserved between human and two other species is shown below the diagonal. The rates of transcription factor binding sites detected in blocks conserved between human and two other species are shown above the diagonal. *Highest BRPR value for these 3-species comparisons.

Most phylogenetic footprinting approaches use evolutionary conservation in order to reduce the search space to the parts of the promoters that are more likely to contain functional *cis*-regulatory elements (for example, see the reports by Sandelin and coworkers [4] and Loots and Ovcharenko [5]). As combinations of more than two genomes are considered, the search space (the jointly conserved region) is reduced. At the same time, the number of sites located within these conserved regions is reduced as well, although at a slower rate. One might then ask, for a given percentage of detectable sites (maximum site sensitivity), which is the combination that minimizes the search space (thereby maximizing specificity)? We found that BRPR scores can be used to address this question. BRPR scores are reversely proportional to *P*(*C*), which is the *a priori *conservation probability (Equation 1; see Materials and methods, below). Thus, the lower the BRPR score, the larger the conserved region and the greater the chance that false-positive TFBS predictions will be made. Therefore, for a given percentage of detectable sites, one wishes to choose the combination of genomes with high BRPR values.

**Figure 4:**
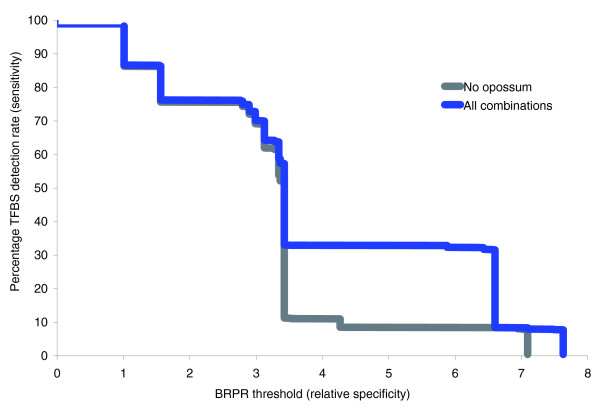
Association between BRPR scores and detectable sites. For each given percent of detectable transcription factor binding sites (TFBSs), the combination of aligned genomes with the highest base regulatory potential rate (BRPR) value will yield the smaller conserved region (for phylogenetic footprinting algorithm searches). The full list of genome combinations and their BRPR values are given in Additional data file 1. The blue line presents the association between percentage of human TFBSs located in conserved regions in a combination of genomes with this BRPR value among all possible genome combinations in this study (see text for detailed description). The grey line plot is similar after the opossum genome is omitted (see text). BRPR, base regulatory potential rate.

We ranked each of the 1,162 tested human TFBSs according to the highest BRPR value from the combinations of genomes that could detect the given site. From this ranking of sites, it may be seen that some subsets of highly conserved TFBSs may be detected at much higher BRPR thresholds than those sites that are conserved only with closely related species. The proportion of TFBSs that may be detected for a given BRPR threshold is plotted in Figure 4 (blue line). This figure shows, for example, that in order to guarantee detection of 75% or more of the known TFBSs, one should choose a combination of genomes with BRPR value of 1.7 or less. Naturally, these will be closely related species. By contrast, the combination of genomes with the overall maximum BRPR score (human-chimp-mouse-opossum-chicken, BRPR 7.628) includes only about 7.7% of the known TFBSs in its conserved regions, whereas the lowest possible BRPR score (human-chimp, BRPR 1.009) includes about 98%. BRPR values may be more appropriate than evolutionary distance for the purposes of weighting contributions when aiming to discover constrained regulatory sequences in multispecies alignments. We therefore suggest that when it comes to regulatory regions, the BRPR score may be more useful that the 'conservation scores' currently employed in phastCons [38] or MCS [39] approaches.

Figure 4 also shows the importance of including the opossum genome in the comparisons. The grey line displays the same graph, but excluding the opossum genome from the plotted combinations. Without including the opossum genome, the BRPR threshold must be reduced to 3.5 before 20% of the known TFBSs may be found in the conserved regions. However, with the opossum included, the BRPR threshold for the same search may be increased to 6.5, indicating analogous reduction in the search space. Figure 4 shows that opossum's greatest contribution in terms of phylogenetic footprinting efficiency is for the sensitivity values in the range of 10% to 33%, although smaller improvements are observed in the 55% to 65% range. The 'blocky' nature of the plot is attributable to the subsets of known TFBSs that are detectable in each of the eight species. As more distant mammalian genomes are sequenced, this plot may smooth out to give higher *P*(*R|C*) scores to more of the known TFBSs.

Our preliminary results including unpublished genomes show that more sites may be predicted with increased BRPR thresholds. Only 20 human sites (1.72% of known TFBSs) are not detected by any combinatorial approach, suggesting that only a small minority of human TFBSs may not be conserved in any other species. It should also be noted that without the chimp genome, a maximum of 86.5% of the sites can be identified as conserved, suggesting that only 13.5% of known human TFBSs may be conserved only among primates. This is an interesting finding, because it establishes 86.5% as an upper limit to the proportion of TFBSs that may be found using traditional phylogenetic footprinting techniques with mouse or more distantly related species. If complete detection of all functional human TFBSs is required, then the phylogenetic shadowing technique for comparing closely related species, proposed by Boffelli and colleagues [40,41], may be more effective than traditional phylogenetic footprinting for primate-specific TFBSs. However, as suggested by those authors, at least six primate genome sequences other than human will be required before phylogenetic shadowing will become effective [40]. Another interesting approach is presented in the recent report by Donaldson and Göttgens [42], which used the mouse genome as an outgroup compared with human and chimpanzee promoters in order to discover regulatory motifs that are conserved in one but not the other [42].

### Exploring dependencies between transcription factor binding site nucleotide conservation and the associated transcription factors

As noted above, the nucleotide conservation within the human TFBSs (as compared with other vertebrates) is higher than the percentage identity in the conserved blocks where they reside (Table 2). This is expected because the regulatory nucleotides may be under stronger evolutionary pressure. Similarly, one would expect that high information content positions (the most conserved positions of the motif) are critical for the binding and thus would also be most conserved across species. This assumption does not take into consideration possible differences in the binding protein residues between species, but it has been shown to be correct for individual yeast and fruit fly transcription factors [43,44]. However, this dependence appears to become weaker when average conservation data are calculated over positions from different vertebrate transcription factors.

**Figure 5:**
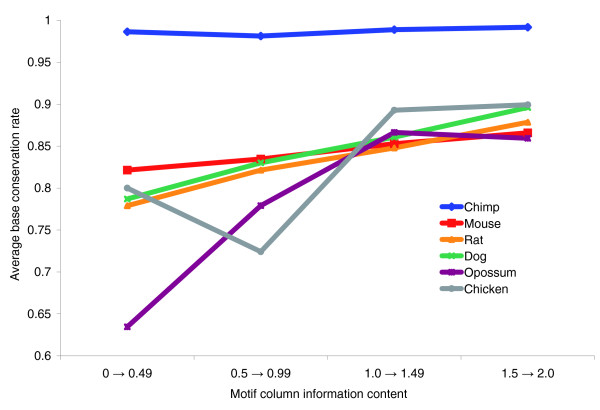
Cross-species conservation of individual TFBS positions versus their information content. Conservation is measured between the human and each of the other species. Information content is measured according to the human position-specific score matrix (PSSM) model.

From the transcription factors included in our dataset, 80 have a position-specific scoring matrix (PSSM) binding model in JASPAR [45] or our manually curated set of mammalian motifs [6,46]. These transcription factors are associated with 544 sites in our dataset. The PSSM model of the corresponding transcription factor was used to scan each of its sites from our dataset (see Materials and methods, below). Sometimes the recorded sites extend beyond the length of the PSSM model, reflecting the biochemical method used to discover these sites (for example, DNA footprinting). The highest scoring (sub)sequence was considered to be the correct target site (TFBS), and conservation of each of its nucleotides was calculated for the species in which the site was conserved. The results are plotted in Figure 5, sorted by information content of the corresponding PSSM columns. A weak but definite trend is present in the nonprimate genomes, although even transcription factor motif positions with zero information content (typically assumed to be under no selective pressure) are conserved at a higher rate than the wider conserved blocks. This finding suggests that natural selection operates almost equally strongly across the TFBS positions, regardless of the perceived role of the nucleotide in protein-DNA interactions. One possible explanation for the observed trends is that some motif positions with lower information content may play an indirect role in DNA binding, perhaps by facilitating DNA conformation or by some other mechanism (for instance, Burden and Weng [47] demonstrated conserved DNA structural features at degenerate TFBS locations).

As noted by Sauer and coworkers [11], for human-rodent comparisons certain transcription factors are more likely to have their TFBSs conserved across species than others. We test this finding outside eutherians by examining conservation rates of TFBSs for those factors for which at least seven instances are detectable in the corresponding comparisons. The findings for human-mouse and human-opossum comparisons are presented in Tables 4 and 5, and similar comparisons between human and other species are available in Additional data file 1.

**Table 4:** Human-mouse TFBS conservation dependency on transcription factor identity

Factor	Motif		Human versus mouse			
		
	IC	Length	Detectable	% conserved	*p *value	Over/under
HMG	8.43	9	7	100.00%	0.1029	
CREB	11.52	8	17	94.12%	0.0257	Over
c-Myb	14.15	11	11	90.91%	0.1186	
NF-AT1	N/A	N/A	10	90.00%	0.1494	
IPF1	N/A	N/A	9	88.89%	0.1862	
p50	15.63	11	8	87.50%	0.2292	
NF-κB	13.34	10	14	85.71%	0.1425	
AhR	8.62	6	7	85.71%	0.2775	
GR	7.06	6	7	85.71%	0.2775	
E2F-1	10.17	8	12	83.33%	0.1982	
AP-1	9.44	7	34	82.35%	0.0686	
HIF-1	11.00	11	11	81.82%	0.2286	
MITF	N/A	N/A	11	81.82%	0.2286	
ATF-2	N/A	N/A	9	77.78%	0.2864	
USF1	10.37	6	9	77.78%	0.2864	
C/EBPα	11.12	9	22	77.27%	0.1745	
p53	25.74	18	22	72.73%	0.1897	
E2F	13.84	8	11	72.73%	0.2631	
c-Ets-1	N/A	N/A	7	71.43%	0.3193	
HNF-1α	N/A	N/A	7	71.43%	0.3193	
Egr-1	13.12	9	12	66.67%	0.2184	
POU1F1a	7.57	5	12	66.67%	0.2184	
Sp1	9.22	8	115	66.09%	0.0250	Under
HNF-1α-A	13.66	10	11	63.64%	0.2010	
GATA-1	5.57	4	14	57.14%	0.1007	
TCF-4	12.54	7	7	57.14%	0.2032	
EBF	21.10	15	8	50.00%	0.1120	
AP-2αA	N/A	N/A	23	47.83%	0.0073	Under
ER-α	N/A	N/A	11	45.45%	0.0405	Under
Crx	11.60	10	7	42.86%	0.0772	
Gfi1	7.60	4	17	35.29%	0.0012	Under*
AR	N/A	N/A	7	14.29%	0.0022	Under

Factors with more than seven sites detectable between the two species are shown. The *p *values given pertain to the observed percentage of conserved sites, and were determined using the Fisher's exact test. Over/under, specifies over-conservation or under-conservation of the sites of the corresponding transcription factor (by Fisher's exact test) at the 5% significance level; *Significant under-representation after *p *value correction (using Bonferroni). Detectable, total number of human transcription factor binding sites located in promoters of mouse orthologous genes; % conserved, percentage of detectable sites that are in conserved regions; IC, information content (total); Length, length of the motif; N/A, there is no available position-specific score matrix model for this transcription factor; TFBS, transcription factor binding site.

**Table 5:** Human-opossum TFBS conservation dependency on transcription factor identity

Factor	Motif		Human versus opossum			
		
	IC	Length	Detectable	% conserved	*p *value	Over/under
HMG	8.43	9	7	100.00%	0.0020	Over*
p50	15.63	11	8	75.00%	0.0470	Over
MITF	N/A	N/A	10	70.00%	0.0487	Over
CREB	11.52	8	13	69.23%	0.0287	Over
E2F-1	10.17	8	10	60.00%	0.1228	
GR	7.06	6	7	57.14%	0.2056	
HNF-1α	N/A	N/A	7	57.14%	0.2056	
POU1F1a	7.57	5	9	55.56%	0.1794	
E2F	13.84	8	11	54.55%	0.1594	
AP-1	9.44	7	24	50.00%	0.1112	
ATF-2	N/A	N/A	8	50.00%	0.2422	
USF1	10.37	6	8	50.00%	0.2422	
IPF1	N/A	N/A	9	44.44%	0.2565	
HIF-1	11.00	11	7	42.86%	0.2938	
p53	25.74	18	16	37.50%	0.1949	
HNF-1α-A	13.66	10	8	37.50%	0.2763	
NF-κB	13.34	10	11	36.36%	0.2321	
Sp1	9.22	8	86	29.07%	0.0049	Under
AP-2αA	N/A	N/A	23	26.09%	0.0581	
C/EBPα	11.12	9	16	25.00%	0.0886	
Egr-1	13.12	9	8	25.00%	0.1961	
c-Myb	14.15	11	11	18.18%	0.0775	
ER-α	N/A	N/A	9	11.11%	0.0521	
GATA-1	5.57	4	9	11.11%	0.0521	
Gfi1	7.60	4	11	0.00%	0.0028	Under
AhR	8.62	6	7	0.00%	0.0238	Under
TCF-4	12.54	7	7	0.00%	0.0238	Under

See Table 5 footnote for details.

Although some factors' TFBSs are conserved at higher than expected (for example, CREB) or lower than expected (for example, Gfi1, AR and Sp1) rates in human-mouse comparisons, only the sites of Gfi1 are (under)conserved after the Bonferroni correction (see Materials and methods, below). Similarly, the sites of various factors are over-conserved (for example, HMG and CREB, among others) and under-conserved (for example, Gfi1 and Sp1, and so on) in human-opossum comparisons, but only the HMG sites remain (over)conserved after the correction (Table 5). We found that all detectable HMG sites are conserved in both mouse and opossum, but their small number (seven) made them appear significant only in the human-opossum comparisons. Interestingly, human Sp1 TFBSs are under-conserved in all genomes except rodents (Additional data file 1). This may be explained by the fact that the Sp1 target site (consensus: 'GGcGGG') and related patterns are expected to occur frequently in GC-rich mammalian promoters. As such, random mutations in mammalian promoters have a high probability of producing additional copies of functional sites. With such a potential proliferation of 'backup' Sp1 target sites, an increased Sp1 TFBS turnover rate should not be surprising. Therefore, evolutionary conservation of TFBSs has some dependency on the identity of the bound transcription factor, but no strong conclusions can be drawn at this point because of the limited amount of available data. AP-2α is represented by 23 human sites in our dataset. All genes regulated by these sites have orthologs in both mouse and opossum, and yet its TFBSs are under-conserved in mouse. This is an example in which TFBS conservation does not coincide with the conservation of the downstream genes, which has been observed for developmental genes as well [1].

We found no association between the information content (IC) of the transcription factor motif and the percentage conservation. For example, TCF-4 motif has a relatively high IC value (12.5) and its sites are generally under-conserved in both mouse and opossum, but they are significantly under-conserved only in opossum (Tables 4 and 5). In contrast, the sites of HMG are all in conserved regions in human-mouse and human-opossum comparisons, yet the HMG motif has an IC value of 8.4.

### Exploring transcription factor binding site conservation dependencies on Gene Ontology categories between human and opossum

We also test the possible association between TFBS turnover rates and the functional category of the corresponding regulated genes. Previous studies suggest that the genes with the highest upstream sequence conservation coverage are those involved in transcription and development [48-51]. Table 6 presents the top 30 most populated GO-slim categories [28] in terms of human-mouse orthologous genes from our 513 protein coding gene dataset. Significance was assessed using the Fisher's exact test, as described in the Materials and methods (below). We found that GO categories 'physiologic process' and 'transporter activity' to be over-represented and under-represented, respectively, in both mouse and opossum, even after the Bonferroni correction. Many other GO categories have over-conserved TFBSs in the promoters of their member genes between human and mouse. Examples include 'transcription', 'development', 'cell-cell signaling', response to various stimuli, among others (Table 6). Sauer and coworkers [11] also showed that TFBS conservation in human-rodent comparisons is correlated with the functional category of the downstream regulated gene. Their findings agree with ours in many categories. In particular, there are 34 categories in common for which one (or both) of the studies has found them to be statistically over-represented or under-represented. In 29 of them (85%) the two studies agree with respect to the 'sign' of conservation. The differences observed between the two studies can be attributed to the different set of TFBSs upon which their measurements are based (Sauer and coworkers used sites from mouse and rat in addition to human) and the methods used to assign significance.

**Table 6:** Human-mouse TFBS conservation dependency on the GO category of the downstream regulated gene

GO category	Number of genes	Upstream coverage	Detectable TFBSs	% TFBS detected	*p *value	Over/under
Transcription regulator activity	34	37.65%	128	83.59%	6.63 × 10^-4^	Over*
Cell-cell signaling	44	26.00%	141	82.27%	1.27 × 10^-3^	Over*
Development	55	35.19%	157	81.53%	1.41 × 10^-3^	Over*
Nucleotide binding	42	23.31%	137	79.56%	1.04 × 10^-2^	Over
Response to biotic stimulus	81	22.67%	273	79.49%	5.62 × 10^-4^	Over*
Response to external stimulus	65	23.49%	209	79.43%	2.56 × 10^-3^	Over
Response to stress	91	23.78%	316	79.11%	3.50 × 10^-4^	Over*
Physiologic process	154	23.59%	526	78.90%	1.37 × 10^-6^	Over*
Cell proliferation	53	29.13%	209	78.47%	6.00 × 10^-3^	Over
Receptor binding	65	24.36%	246	77.24%	9.74 × 10^-3^	Over
Receptor activity	42	24.55%	114	77.19%	4.29 × 10^-2^	Over
Mitochondrion organization and biogenesis	100	25.26%	266	77.07%	8.93 × 10^-3^	Over
Transcription	67	35.72%	223	76.68%	1.82 × 10^-2^	Over
Extracellular region	56	21.66%	217	76.04%	2.73 × 10^-2^	Over
Protein binding	142	26.43%	464	75.86%	4.75 × 10^-3^	Over
Extracellular space	54	23.08%	232	75.86%	2.70 × 10^-2^	Over
Regulation of biologic process	155	29.96%	562	75.27%	4.97 × 10^-3^	Over
Cytoplasm	45	22.87%	136	74.26%	7.17 × 10^-2^	
Plasma membrane	57	20.12%	143	74.13%	7.10 × 10^-2^	
Transcription factor activity	42	36.92%	137	73.72%	7.62 × 10^-2^	
Nucleus	92	31.28%	332	73.49%	5.00 × 10^-2^	
Cell death	48	21.97%	189	73.02%	6.95 × 10^-2^	
Protein metabolism	49	19.65%	147	72.79%	7.83 × 10^-2^	
Biologic process	35	21.69%	100	72.00%	9.24 × 10^-2^	
Signal transduction	116	23.96%	398	71.86%	5.33 × 10^-2^	
Cell cycle	41	28.45%	182	70.88%	6.34 × 10^-2^	
Cell	118	21.23%	351	69.23%	1.68 × 10^-2^	Under
Binding	90	24.17%	297	68.69%	1.58 × 10^-2^	Under
Transport	39	24.11%	146	67.81%	3.30 × 10^-2^	Under
Catalytic activity	40	19.68%	99	61.62%	4.63 × 10^-3^	Under
Transporter activity	35	25.00%	123	60.98%	1.20 × 10^-3^	Under*

The top 31 Gene Ontology (GO) categories in terms of gene numbers in the dataset are shown. The *p *values given represent the significance (uncorrected) of the observed percentage of conserved (detected) sites, as determined using the Fisher's exact test. Over/under, specifies over-conservation or under-conservation of the sites of the corresponding GO category (by Fisher's exact test) at the 5% significance level. *Statistical over-representation or under-representation after *p *value correction (using Bonferroni). TFBS, transcription factor binding site.

We extend this study in opossum (Table 7) and the other vertebrate genomes (Additional data file 1). Most of the over-conserved categories between human and mouse are also over-conserved in human-opossum comparisons (Fisher's exact test; see Tables 6 and 7). These include 'cell-cell signaling' and response to stress and biotic stimuli. On the other hand, the TFBS conservation rate for the 'protein binding' went from being over-conserved in human-mouse comparisons (76% TFBS conservation) to under-conserved in human-opossum comparisons (36% TFBS conservation). This is one of the highly populated categories, and its members are involved in almost every cellular process, for instance signal transduction, chromatin structure, transcription, translation, cell cytoskeleton, and so on. It is therefore difficult to assess the significance of this change in TFBS conservation related to this category. One thing is for sure; the observed differences are not an artifact caused by the low number of TFBSs. This category is represented by 142 genes associated with 464 TFBSs in mouse and 122 genes associated with 419 TFBSs in opossum, making it one of the best represented categories in our dataset.

**Table 7:** Human-opossum TFBS conservation dependency on the GO category of the downstream regulated gene

GO category	Number of genes	Upstream Coverage	Detectable TFBSs	% TFBS Detected	*p *value	Over/under
Receptor binding	51	6.49%	180	55.56%	5.80 × 10^-6^	Over*
Cell-cell signaling	35	6.37%	120	51.67%	3.67 × 10^-3^	Over
Physiologic process	122	5.63%	415	49.40%	1.51 × 10^-6^	Over*
Response to external stimulus	54	5.60%	168	48.81%	6.12 × 10^-3^	Over
Transcription regulator activity	32	10.15%	122	47.54%	2.47 × 10^-2^	Over
Extracellular space	43	4.08%	175	47.43%	1.23 × 10^-2^	Over
Response to biotic stimulus	60	5.29%	209	47.37%	7.82 × 10^-3^	Over
Transcription	61	10.52%	208	45.67%	2.13 × 10^-2^	Over
Transcription factor activity	40	9.80%	133	45.11%	4.65 × 10^-2^	Over
Development	47	9.48%	120	45.00%	5.25 × 10^-2^	
Signal transduction	86	5.72%	293	44.71%	1.95 × 10^-2^	Over
Response to stress	74	6.23%	268	44.03%	3.18 × 10^-2^	Over
Regulation of biologic process	134	8.49%	490	43.06%	2.59 × 10^-2^	Over
Cell	82	6.11%	241	40.66%	5.96 × 10^-2^	
Nucleus	81	10.05%	305	40.66%	5.52 × 10^-2^	
Extracellular region	44	6.17%	160	40.63%	6.95 × 10^-2^	
Cell proliferation	49	7.63%	196	40.31%	6.26 × 10^-2^	
Mitochondrion organization and biogenesis	77	6.90%	213	39.44%	5.29 × 10^-2^	
Cytoplasm	34	6.07%	97	39.18%	7.95 × 10^-2^	
Cell death	41	6.77%	164	37.80%	4.34 × 10^-2^	Under
Protein binding	122	7.01%	419	35.80%	4.81 × 10^-4^	Under*
Cell cycle	39	7.67%	176	35.23%	1.35 × 10^-2^	Under
Nucleotide binding	32	5.82%	112	31.25%	5.81 × 10^-3^	Under
Protein complex	28	5.90%	84	29.76%	7.37 × 10^-3^	Under
DNA binding	27	10.05%	74	29.73%	1.08 × 10^-2^	Under
Binding	71	6.77%	240	29.58%	5.58 × 10^-6^	Under*
Receptor activity	31	6.53%	88	29.55%	5.67 × 10^-3^	Under
Plasma membrane	37	4.78%	91	28.57%	3.00 × 10^-3^	Under
Protein metabolism	41	6.27%	131	25.95%	3.75 × 10^-5^	Under*
Transporter activity	31	6.28%	91	23.08%	6.74 × 10^-5^	Under*
Transport	32	5.53%	102	20.59%	1.85 × 10^-6^	Under*

See Table 6 footnote for details.

'Development' is another category in which TFBSs are significantly over-conserved in human-mouse comparisons. In human-opossum comparisons TFBSs are still over-conserved, but not at a significant level. This can also be attributed to the sharp decrease in the percentage of detected TFBSs (from 81.5% in mouse to 45% in opossum) in relation to the high number of potentially detectable TFBSs (157 versus 120 in mouse and opossum, respectively). The developmental genes themselves are ultra-conserved in opossum [1], resulting in the detection of many orthologs and hence many potentially detectable TFBSs associated with them. Conservation tables, similar to Tables 6 and 7, for comparisons between human and other species are available in Additional data file 1.

### Comparison with other studies

A number of existing studies have attempted to quantify regulatory conservation in mammals, albeit using different approaches and typically restricting their interest to human-rodent comparisons. Our results on human-rodent comparisons generally agree with these studies. For example, we find approximately 72% of detectable human TFBSs conserved in mouse 5 kb upstream regions. Similarly, Sauer and coworkers [11] reported detection of TRANSFAC [29] TFBSs in human-rodent conserved sequences at a rate of 71.7% when using the same conservation threshold (65% identity).

For conservation cutoffs of 70% identity, Liu and coworkers [9], Levy and Hannenhalli [12], and Lenhard and colleagues [13] independently found human-mouse conservation rates for known TFBSs of about 60%, 65%, and 68%, respectively. The latter three studies were also based on finding conserved blocks via sliding windows on aligned sequences. Dermitzakis and Clark [10] also reported detection of TRANSFAC TFBSs in human-rodent conserved sequences at rates of 60% to 68%. All of the aforementioned human-rodent TFBS turnover rates are consistent with our findings, given the slightly higher conservation cut-offs and the lower number of known TFBSs tested (40 sites by Lenhard and colleagues [13], 64 sites by Dermitzakis and Clark [10], 467 sites by Liu and coworkers [9], and 481 sites by Levy and Hannenhalli [12]).

In relation to our human-mouse 5 kb upstream conservation coverage figure (24%), a number of other studies have found human-rodent upstream conservation rates in the range 17% to 25% [9,52,53]. In a comparison of 77 well defined human-mouse gene pairs, Jareborg and coworkers [54] found 36% conservation coverage of upstream sequence using the software program DBA and a 60% cutoff. However, their upstream sequences ranged from 500 bp to 1,000 bp upstream of the TSS. Our conservation coverage in the same range of distance is 38.7% to 49.2%. Sauer and coworkers [11] found a background conservation rate of 35% in human-rodent comparisons, although their study was based on 800 bp windows of sequence centered on a known TFBSs, and was therefore also biased toward including sequence from the proximal 500 bp region.

A recent study of the mouse transcriptome showed that a large part of this mammalian genome may be transcribed [55]. The authors found many more transcripts than the number of genes currently estimated for the mammalian genomes. For about one-third of these transcripts no association with protein coding genes was found, and therefore they were considered to be noncoding RNAs (ncRNAs). Similar to our study, the authors analyzed the upstream sequences of these potential ncRNAs, which they found to be more conserved than the promoters of the protein coding genes. However, their study has some differences compared with ours. First, it does not focus specifically on the intergenic miRNA genes, but analyzes all transcripts for which no protein coding gene association was found. Also, their study does not depict the similarity we found in the conservation rates of coding and noncoding upstream regions in the first 500 bp, which is an important finding of our study, especially when compared with the conservation of the upstream sequences of the tRNA genes (Figure 2). Cooper and coworkers [56] recently analyzed the conservation rates of core promoter sequences of protein coding genes. Their findings agree with ours in that they find that the first 300 bp upstream of the TSS are important for the core promoter activity. This is the region where we find the highest conservation (Figure 2). In another study, Taylor and coworkers [57] reported that the nucleotide substitution rate increases with the distance from TSS in various types of protein coding genes in a way similar to our observations.

## Conclusion

This study is the first to analyze conservation of the upstream regions of protein coding genes in relation to the upstream regions of intergenic miRNA genes. We found the latter to be about twice as conserved as the former beyond the first 500 bp. The reason for this conservation is currently unknown. The first 500 bp appear to be equally conserved in both types of genes, a feature that is missing from the upstream sequences of the tRNA genes. This indicates that similar mechanisms of gene regulation may be in place, which is in agreement with other studies [27,32]. The difference in conservation rates is more apparent in the mammalian lineages, including opossum, and may reflect similarities in mammalian gene regulation.

Another important finding is that the opossum genome offers great potential in terms of improving the performance of the phylogenetic footprinting algorithms. We found that 41% of the known human TFBSs are located in the 6.7% of promoter regions that are conserved between human and opossum, illustrating that the opossum genome sequence can be used to reduce the search space for a large proportion of human TFBSs. A new statistical measure, BRPR, is introduced that quantifies the trade-off between sequence conservation (or reduction of the search space for comparative genomics strategies) and regulatory site conservation. We show that for a given site sensitivity threshold, an appropriate combination of genomes can be selected to minimize the search space. Finally, we find that basic cellular functions, such as cell-cell signaling and receptor binding, have significantly over-conserved sites between human and opossum (the corresponding genes have more TFBSs located in the conserved parts of their promoter regions). By contrast, TFBSs related to functions such as transporter activity and protein metabolism are significantly under-conserved.

## Materials and methods

### MicroRNA gene dataset

Human miRNA genes were retrieved from the miRBase [58] and the UCSC Genome Browser (version hg18, March 2006) [59]. Cross-referencing them with the miRNAMap dataset [60] identified 169 putatively intergenic miRNA genes. The sequences of these miRNAs were used in BLAST-like Alignment Tool (BLAT) [61] alignments against the latest UCSC human genome and their exact genomic locations were identified. Following observations in previous studies [27,62], we consider two miRNA genes to be co-transcribed if their starting points are less than 250 bp apart. In this way, we identified 12 clusters containing 31 genes. Only the 5'-most gene in a cluster was considered in our analysis. Five miRNA genes were found to reside within large introns of protein coding genes, and although they may have their own regulatory regions, we excluded them from further analysis. This resulted in a dataset of 145 human intergenic miRNA genes (Additional data file 1). The coordinates of the BLAT outputs were used to retrieve up to 5 kb regions upstream of the gene start site as described below.

We note that in a recent study, Devor and Samollow (personal communication) tested 71 predicted miRNA genes using quantitative polymerase chain reaction on pooled RNA from brain, heart, lung, liver, tongue, and esophagus from an adult opossum. They found evidence of expression in 80% of the cases they tested, including 36 genes in our opossum dataset.

### Pair-wise and multiple species comparisons

Pair-wise and multiple species alignments for both protein coding and miRNA genes were retrieved from the 17-species MULTIZ multiple alignments [38], which are available from the UCSC web server (version hg18, March 2006) [63]. The MULTIZ algorithm builds a multiple alignment from local pair-wise BLASTZ alignments of the reference genome with each other genome of interest [38,64]. Each base in the reference genome is aligned to at most one base in the other genomes, and the alignment is guided by synteny. In this study, we present the results from pair-wise and multiple species comparisons of human [65] with four eutherian mammals (chimpanzee [66], mouse [67], rat [68], and dog [15]), the newly sequenced opossum [1], chicken [69], fugu [70], and tetraodon [71]. A phylogenetic tree for those species and with branch lengths derived from the ENCODE project Multi-Species Sequence Analysis group (September 2005) is shown in Figure 1. This tree was generated using the phyloGif program [72] from Threaded Blockset Aligner (TBA) alignments over 23 vertebrate species and is based on 4D sites (similar to the tree presented by Margulies and coworkers [73]).

For each pair-wise or multiple species comparisons, the corresponding (aligned) 5 kb upstream sequences were retrieved directly from the MULTIZ alignments for greater accuracy, using the human genes as reference. If other genes were found within this 5 kb range, then the upstream sequences were shortened accordingly to exclude the additional genes. We used the 65% as our conserved block threshold, which is similar to that in previous studies [9,12,13] and similar to the default threshold used by many phylogenetic footprinting algorithms [6,13].

### tRNA dataset

Human tRNA genes and pair-wise alignments were extracted from the UCSC Genome Browser database (version hg18, March 2006) using the genomic MULTIZ alignments as we describe above. Genes that were found to be facing opposite directions in the genome ('head-to-head') and their starts were closer than 2.5 kb apart were excluded from the analysis. This rule excluded 156 genes. The final human tRNA dataset included 1,795 upstream sequences.

### Dataset of known transcription factor binding sites

TRANSFAC database (release 9.3) [29] contains 1,162 human confirmed TFBSs that satisfy the following criteria: the site is experimentally confirmed and associated with a promoter of a human gene from the database (confirmed sites); the TFBS sequence can be found within 5 kb upstream of the TSS; if multiple site occurrences are present in the corresponding promoter, then positional information (relative to TSS) is listed in the database; and the regulated human gene corresponds to an entry in the RefSeq gene collection. The above TFBSs are located in the promoters of 513 human genes, which serves as our primary dataset for the transcription factor-TFBS association study. We focus on the sites located in the 5 kb upstream region, because this includes 83.4% of all known human TFBSs in TRANSFAC (data not shown). The majority of the sites (a total of 774) have a TRANSFAC assigned quality score of 1, 2, 3, or 4, which shows confirmed binding activity to a known transcription factor. For an additional 325 sites, no TRANSFAC quality score was assigned. The remaining 63 sites (about 5%) belong to TRANSFAC category 5, for which an unknown protein has been shown to bind to a DNA element.

### Dataset of position-specific scoring matrix models

JASPAR database [35] contains 20 PSSM models for transcription factors whose sites are present in our dataset. In addition, we previously generated manually 60 more PSSM models from high-quality human and mouse sites in TRANSFAC [6], which we make publicly available through our web server [74]. These models were used to analyze the position information content with the nucleotide conservation in the subset of 572 corresponding known TFBSs (Figure 5).

### Conserved blocks and transcription factor binding site detection: some definitions

In this study, sequence conservation is expressed as conserved block coverage. A sliding window of width 50 bp and step size 10 bp was used to find conserved regions (or blocks) of at least 65% identity between human and each other species. Each pair-wise alignment was extracted from the MULTIZ multiple alignments. Sauer and coworkers [11] have shown that the 65% identity threshold most effectively separates TFBSs from background sequence in human-rodent comparisons. The percentage of human 5 kb upstream sequence that is located within conserved blocks is denoted the 'conserved block coverage'. The 'average block conservation' is the percentage of identical bases in conserved blocks over all bases in conserved blocks. A 'conserved site' is a known human TFBS that overlaps a conserved block between human and another species. Because we explore the effect of sequence and pattern of conservation in the discovery of *cis*-regulatory elements, this study does not make any assumptions about the biologic functionality of the human-equivalent TFBSs in the other organisms. In other words, we cannot address the issue of actual site turnover, but simply whether a known human TFBS is located in a conserved block between human and one or more other species (regardless of whether it is functional in these other species). 'Detectable TFBSs' are those sites that are in the promoters of genes that have orthologs in the other species (in terms of UCSC multispecies alignments). A detectable site is considered to be 'conserved' between two species if it is located in a conserved block in their corresponding pair-wise alignment. When multiple species are considered, a TFBS is considered to be conserved if it is conserved in each of the species. The 'TFBS conservation rate' between human and other species is defined as the percentage of detectable TFBSs found to be conserved. The conservation rate can be thought of as the upper limit of sensitivity (at the site level) of a phylogenetic footprinting algorithm if only the conserved regions are analyzed. Such algorithms include ConSite and rVista [13,75]. In general, the methods and thresholds used to define conserved blocks were chosen to reflect those typically used by phylogenetic footprinting algorithms [6,13,46,75] and by other researchers [9,11-13].

### Base regulatory potential rate

A base position is called 'regulatory' if it is part of a TFBS. For this report, bases in nonhuman species that are aligned to human regulatory bases are also called regulatory. We understand that this definition is only made for the purposes of this analysis and does not imply any functional role. However, it is expected that the majority of known human sites that are conserved in various species would also be functional in these species. Given a promoter alignment between two species, we define the base regulatory potential rate (BRPR) as the conditional probability of a base being regulatory given it is located in a conserved region over the prior probability of being regulatory. Formally, BRPR is defined in the first part of the following equation:

BRPR=P(R|C)P(R)=P(C|R)P(C)
 MathType@MTEF@5@5@+=feaafiart1ev1aaatCvAUfeBSjuyZL2yd9gzLbvyNv2Caerbhv2BYDwAHbqedmvETj2BSbqee0evGueE0jxyaibaiKI8=vI8tuQ8FMI8Gi=hEeeu0xXdbba9frFj0=OqFfea0dXdd9vqai=hGuQ8kuc9pgc9s8qqaq=dirpe0xb9q8qiLsFr0=vr0=vr0dc8meaabaqaciGacaGaaeqabaqadeqadaaakeaacaqGcbGaaeOuaiaabcfacaqGsbGaeyypa0ZaaSaaaeaacaWGqbGaaiikaiaadkfacaGG8bGaam4qaiaacMcaaeaacaWGqbGaaiikaiaadkfacaGGPaaaaiabg2da9maalaaabaGaamiuaiaacIcacaWGdbGaaiiFaiaadkfacaGGPaaabaGaamiuaiaacIcacaWGdbGaaiykaaaaaaa@4830@

where *R *denotes the base as regulatory (part of a known human TFBS) and *C *indicates that it is located in a conserved region. The last part of the equation derives from the Bayesian rule and is the one we use for the calculation of BRPR because *P*(*R|C*) cannot be reliably estimated, given our limited knowledge of mammalian TFBSs. In other words, BRPR shows how much we improve our regulatory potential prediction if we restrict our search space to conserved regions only. *P*(*C*) and *P*(*C|R*) are directly estimated from the data. *P*(*R*) is the *a priori *probability of a base being regulatory in a given promoter, and it depends on the size of the promoter as well as the number and size of *cis*-regulatory elements found within. According to our current knowledge of transcriptional control, *P*(*R*) decreases as one examines windows of sequence more distal to the transcription start site. In this way, calculated BRPR values are dependant on the length of upstream sequence examined from the transcription start. BRPR values decrease as the examined regions become smaller (5 kb to 1 kb or 500 bp from the TSS; Additional data file 1 [Supplementary Figure 1]) because, from Equation 1 above, *P*(*R*) increases in these shorter regions while *P*(*R|C*) remains relatively constant. The important point to note, however, is that the relative BRPR rankings of different genome combinations remain constant (Additional data file 1 [Supplementary Figure 1]).

### Assessing significance of over-conservation or under-conservation for sets of transcription factor binding sites

The Fisher's exact test on 2 × 2 contingency tables is used to estimate the significance of under-conservation or over-conservation of sites bound by particular transcription factors or associated with certain GO categories (Tables 4 to 7). To account for multiple testing we applied the Bonferroni correction, although the data dependencies among the tests make that correction slightly conservative. Statistically over-represented and under-represented categories are presented in Tables 4 to 7 in the corresponding column, and those values that remain significant after the Bonferroni correction are marked with asterisks.

## Additional data files

The following additional data are available with the online version of this paper. Additional data file 1 provides various descriptions, generalized analyses, and supplementary data that complement and extend those given in the main text.

## Supplementary Material

Additional data file 1 Supplementary Text 1 describes the dependence of conservation rates on the methods employed. Supplementary Text 2 provides a note on some further properties of the BRPR score. Supplementary Figure 1 illustrates the behavior of BRPR scores in mammalian comparisons as the window of examined upstream sequence is reduced. Supplementary Figure 2 reproduces some of the information in Figure 2 (main text), but includes error bars in order that statistical significance of our analysis may be judged. Supplementary Table 1. A shows conservation rates of 5 kb upstream regions and TFBSs as found by the DNA Block Aligner (DBA)-based analysis. Supplementary Table 1. B shows conservation rates of 5 kb upstream regions and TFBSs, as found by the UCSC multiple alignment-based analysis. Supplementary Tables 2 to 9 show TFBS conservation dependency on transcription factor identity for human sites conserved in other species (based on UCSC multiple alignment analysis). Supplementary Tables 10 to 17 show TFBS conservation in relation to the GO category of the regulated gene for human sites conserved in eight other species (based on UCSC multiple alignment analysis). Supplementary Table 18 provides conservation rates of 5 kb upstream regions and TFBSs for human compared with 218 combinations (_8_C_5_) of the eight other tested genomes (based on UCSC multiple alignment analysis). Supplementary Table 19 provides a re-analysis of 5 kb upstream coverage rates and regulatory site conservation using only those sites/regulated genes stored in TRANSFAC public (v. 7.0). Click here for file
