# Supplementary material for: Regulatory conservation of protein coding and microRNA genes in vertebrates: lessons from the opossum genome
Source: Genome Biol. 2007 May 16;8(5):R84. doi: 10.1186/gb-2007-8-5-r84 (PMC1929153; doi:10.1186/gb-2007-8-5-r84)
Supplement: Additional data file 1 — Supplementary Text 1 describes the dependence of conservation rates on the methods employed. Supplementary Text 2 provides a note on some further properties of the BRPR score. Supplementary Figure 1 illustrates the behavior of BRPR scores in mammalian comparisons as the window of examined upstream sequence is reduced. Supplementary Figure 2 reproduces some of the information in Figure 2 (main text), but includes error bars in order that statistical significance of our analysis may be judged. Supplementary Table 1. A shows conservation rates of 5 kb upstream regions and TFBSs as found by the DNA Block Aligner (DBA)-based analysis. Supplementary Table 1. B shows conservation rates of 5 kb upstream regions and TFBSs, as found by the UCSC multiple alignment-based analysis. Supplementary Tables 2 to 9 show TFBS conservation dependency on transcription factor identity for human sites conserved in other species (based on UCSC multiple alignment analysis). Supplementary Tables 10 to 17 show TFBS conservation in relation to the GO category of the regulated gene for human sites conserved in eight other species (based on UCSC multiple alignment analysis). Supplementary Table 18 provides conservation rates of 5 kb upstream regions and TFBSs for human compared with 218 combinations (8C5) of the eight other tested genomes (based on UCSC multiple alignment analysis). Supplementary Table 19 provides a re-analysis of 5 kb upstream coverage rates and regulatory site conservation using only those sites/regulated genes stored in TRANSFAC public (v. 7.0). [file gb-2007-8-5-r84-S1.doc]

**Supplementary Information for “Regulatory conservation of protein coding and miRNA genes in vertebrates: lessons from the opossum genome”**

Shaun Mahony^1^, David L. Corcoran^2^, Eleanor Feingold^2,3^, Panayiotis V. Benos^1,2,4^

*^1^ Department of Computational Biology, School of Medicine*

*^2^ Department of Human Genetics, Graduate School of Public Health*

*^3^ Department of Biostatistics, Graduate School of Public Health*

*^4^ University of Pittsburgh Cancer Institute, School of Medicine, University of Pittsburgh, Pittsburgh, PA, USA*

**Table of Contents:**

- *Supplementary Text 1:* Dependence of conservation rates on the methods employed
- *Supplementary Text 2:* A note on some further properties of the BRPR score
- *Supplementary* ***Figure S1****:* The behavior of BRPR scores in mammalian comparisons as the window of examined upstream sequence is reduced.
- *Supplementary* ***Figure S2****:* This figure reproduces the plots in the main manuscript’s Figure 2 for protein coding and miRNA upstream sequence conservation, but includes error bars in each point.
- *Supplementary* ***Table S1.A****:* Conservation rates of 5Kbp upstream regions and TFBSs as found by the DBA-based analysis.
- *Supplementary* ***Table S1.B****:* Conservation rates of 5Kbp upstream regions and TFBSs as found by the UCSC multiple alignment-based analysis.
- *Supplementary* ***Tables S2 – S9****:* TFBS conservation dependency on TF identity for human sites conserved in other species (based on UCSC multiple alignment analysis).
- *Supplementary* ***Tables S10 - S17****:* TFBS conservation in relation to the GO category of the regulated gene for human sites conserved in 8 other species (based on UCSC multiple alignment analysis).
- *Supplementary* ***Table S18****:* Conservation rates of 5Kbp upstream regions and TFBSs for human compared with 218 combinations (_8_C_5_) of the 8 other tested genomes (based on UCSC multiple alignment analysis).
- *Supplementary* ***Table S19****:*Reanalysis of 5Kbp upstream coverage rates and regulatory site conservation using only those sites/regulated genes stored in TRANSFAC public (v. 7.0).
- *Supplementary* ***Table S20****:*List of intergenic miRNA genes used in the analysis.

***Dependence of conservation rates on the methods employed***

Sauer, *et al.* have already evaluated a wide variety of alignment algorithms in the context of phylogenetic footprinting, and have demonstrated the near equivalence of all methods they tested [11]. Nevertheless, we performed an independent study on the same dataset as that used in the main manuscript, but using different analysis methods. Reciprocal BLAST best hits were used to identify the human gene orthologues in other species. Transcription start site (TSS) annotations were automatically extracted from EnsEMBL and local alignments of conserved sequence blocks were calculated with the DNA Block Aligner (DBA) program [54] (65% similarity threshold). This analysis would be expected to give lower quality results than the UCSC multiple alignments based approach presented in the main text, since (a) identification of orthologous genes based on similarity *and* synteny are expected to be more accurate than reciprocal best hits, and (b) automatically annotated TSSs are expected to be less accurate than curated ones. However, the DBA-based results are similar in terms of coverage and TFBS turnover rates to those obtained in the main text. Slightly lower rates of TFBS conservation are observed in many species in the DBA-based study, although these were typically found to be attributable to TSS misannotation (see below).

Note that the use of a sliding window and threshold reduces the amount of sequence that counted as conserved. For example, we found that only ~6.5% of the human-opossum 5 Kbp upstream alignments passes the 65% conservation threshold and 50bp minimum size, whereas when the overall conservation is measured, 22.75% of the human 5 Kbp upstream sequence is aligned with the opossum. This is similar to the proportion found by Margulies, *et al.* in a comparison of 1.9Mbp [73].

*Dataset of known TFBSs*

The TRANSFAC database (Release 9.3) [29] of 2326 human TFBSs associated with 585 genes was filtered as described in the main text. The filtering rules retain only those TRANSFAC entries for which: a) the associated (regulated) gene is listed and can be found in the database, b) the TFBS sequence is listed and is present in the 5Kbp upstream region of the associated gene, and c) positional information (relative to TSS) is listed if the provided TFBS sequence is not unique in the appropriate upstream region.

*Finding homologous upstream regions*

The entire set of protein-coding sequences and their corresponding 5Kbp upstream regions were downloaded from Ensembl (release 39) for each of the following genomes: human, chimpanzee, mouse, rat, dog, opossum, chicken, frog, and zebrafish. For each of the 585 human genes associated with one or more confirmed TFBSs in TRANSFAC, the corresponding protein sequence was extracted from the Ensembl sets. Each protein sequence was BLASTed against all annotated proteins in each of the other genomes under test. Homology was assumed if a *reciprocal best hit* (***RBH***) existed.

Supplementary Table S1.A shows the number of proteins for which RBH existed for different pairs of organisms. Reciprocal best hits are expected to be an accurate signifier of homologous proteins in comparisons of vertebrate genomes, although we recognize that due to gene duplications and inconsistent gene annotation in one or both species, some homologous gene pairs may not be recognized by RBH searches alone. For example, only 480 of the 585 human genes had RBH matches in the chimp genome, although it is well-known that almost every human gene has a chimp ortholog. The failure of the automatic RBH method to find unambiguous chimp orthologs for some human genes is most often explained by the relatively incomplete gene annotation of the chimp genome at the time of this study. For our purposes, however, restricting our focus to those genes for which unambiguous RBHs exist is an acceptable strategy, since all results will be quoted with respect to the sets of orthologous sequence pairs definable for each pair of organisms. Note that since only a subset of genes is homologous between two genomes, the numbers of TFBSs that are possibly *detectable* between two species are also subsets of the original TRANSFAC filtered set. The numbers of detectable sites for each pair of genomes is also shown in Supplementary Table S1.A.

*Alignment and site detection*

In this confirmatory study, the DNA Block Aligner (DBA [54]) was used to find local alignments of conserved sequence blocks in the 5Kbp upstream regions of human genes. DBA was chosen in order to reflect the use of local alignment strategies in typical phylogenetic footprinting applications, and indeed, DBA itself is employed by some popular phylogenetic footprinting software programs [6].

Ensembl annotation was used to define the gene start positions (and therefore the upstream regions). In this study, we make the assumption that the transcription start sites (TSS) are correctly annotated for the set human genes examined. We therefore extract 5Kbp of sequence upstream from the annotated human TSS positions. However, Ensembl gene start positions do not always describe transcription start sites for homologous genes in other species. Even if TSS is implied by the annotated gene start, TSS turnover and annotation inaccuracies may mean that upstream sequences taken from the annotated gene starts in two species do not always accurately reflect homologous genome regions. In order to combat this issue of “TSS-skew”, the human 5Kbp upstream regions were aligned (using DBA) against a region 50Kbp upstream and 5Kbp downstream of the annotated gene start for the homologous genes.

The DBA parameters were adjusted to allow the detection of conserved blocks with a minimum of 65% identity (block opening probability = 0.05). DBA reports a set of conserved “blocks” in the pair of input sequences. *Coverage* rates thus refer to the percentage of human 5Kbp upstream regions that are overlapped by DBA blocks. If a known TFBS is located in a region covered by a conserved block, it is assumed to be conserved between the two genomes (i.e. *detected*), although conservation obviously doesn’t confirm the functionality of a TFBS.

*Results*

Supplementary Table S1.A. shows coverage rates, TFBS detection rates, and average identity rates (weighted by the lengths of the blocks/TFBSs) for each species tested. Supplementary Table S1.B. reproduces the conservation rates found by the UCSC multiple alignment-based approach (as described by Tables 1 & 2 in the main text) to allow ease of comparison.

By comparing Supplementary Tables S1.A & S1.B, it may be seen that both methodologies result in equivalent findings. Compared with the UCSC multiple alignment based approach, higher rates of 5Kbp upstream region coverage are observed in the DBA-based analysis for human compared with mouse, rat, opossum and chicken. However, these increased rates may reflect the increased likelihood that the DBA-based analysis will align repetitive regions in the human 5Kbp upstream region with repetitive, but not necessarily homologous, sequence in the other species (as the 5Kbp human sequence is compared with 55Kbp in the other species). Slightly decreased rates of TFBS conservation are observed in the DBA-based analysis for human compared with other mammalian genome, and this small difference may reflect the greater accuracy of the UCSC multiple alignments over the DBA-based approach. The correlation between TFBS conservation rates in Supplementary Tables S1.A and S1.B seems to break down for human compared with non-mammalian vertebrates, but again, this may reflect shortcomings of the DBA-based approach (including the reliance on Ensembl gene annotation) that are avoided in the UCSC multiple alignments based approach.

Overall, the DBA-based analysis supports the conclusions of the UCSC multiple alignments-based analysis, even though the methodologies are independent. This supports our hypothesis that the conclusions presented in the main text are robust to changes in alignment methodology.

***A note on some further properties of the BRPR score***

*BRPR scores depend on the amount of upstream sequence examined:* The window of upstream sequence examined in the main manuscript is 5 Kbp upstream of every gene. One could ask if the observed BRPR scores for the different genome combinations depend on this definition of “upstream sequence”. For example, would the same scores be observed if we had examined only 1 Kbp upstream of each gene? In order to explore this, let us first remind ourselves of Equation 1:

.

We know from Figure 2 in the main manuscript that *p(C)* increases as one approaches the TSS in the average protein-coding gene. However, we do not observe a corresponding increase in the proportion of regulatory regions that are conserved (*p(C|R)*) as we approach the TSS. Therefore, from the second half of the above equations, the BRPR value for a given combination of genomes will decrease as the window of examined upstream sequence is made smaller. This, indeed, is what we observe in *Supplementary Figure 1* below. The important thing to observe is that even with this dependence of the BRPR score on the amount of upstream sequence examined, the *relative effectiveness* of one genome combination in relation to another remains unchanged. In other words, our conclusions about the most effective phylogenetic footprinting strategies remain valid in the face of changes to the upstream region examined, even if the actual BRPR scores observed change.

*BRPR values in Supplementary Table S16 and sampling errors:* Note that some higher order multiple species combinations may have lower BRPR values than combinations with subsets of these species. This is expected to occur mainly when a combination includes the chicken or a fish genome and it is due to the small values of *p(C|R)* and *p(C)* estimates that can lead to large errors. For example, the combination of chimp and chicken results in a lower BRPR value (*BRPR* = 5.845) than using chicken alone (*BRPR* = 6.184). Of course, all bases conserved between human, chimp and chicken will also be conserved between human and chicken. In cases such as this, the higher BRPR value is the one reported.

**Figure S1:** The behavior of BRPR scores in mammalian comparisons as the window of examined upstream sequence is reduced.

**
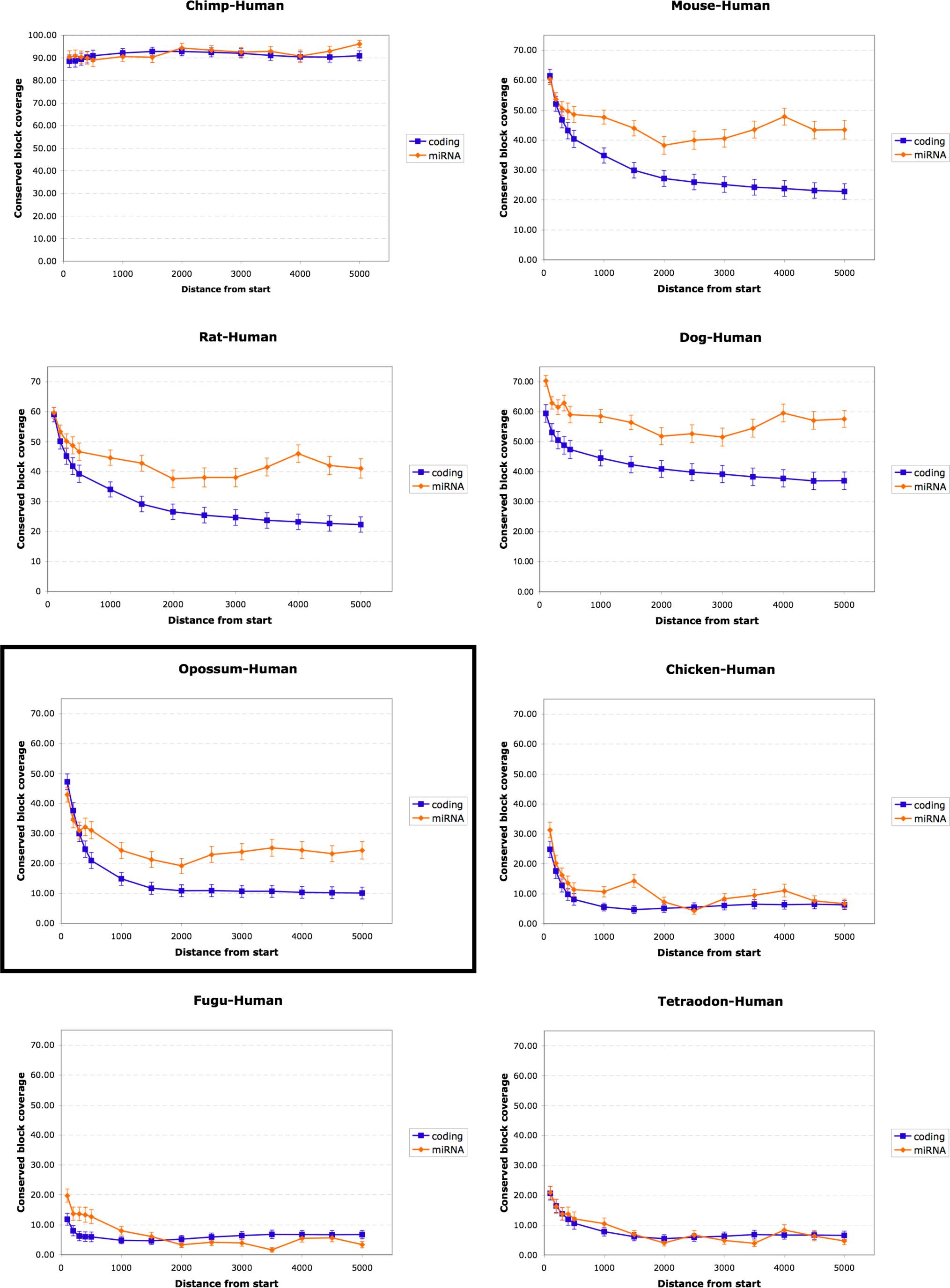
**

**Supplementary Figure S2:** This figure reproduces the plots in the main manuscript’s Figure 2 for protein coding and miRNA upstream sequence conservation, but includes error bars in each point.

**Supplementary Table S1.A.** Conservation rates of 5Kbp upstream regions and TFBSs as found by the DBA-based analysis.

| **Human genes vs.** | ***RBH gene pairs*** | ***Block Coverage*** | ***Avg. Block Identity*** | ***Detectable Sites*** | ***TFBSs Detected*** | ***Avg. Conserved Site Id.*** |
| --- | --- | --- | --- | --- | --- | --- |
| *Chimp* | 480 | 89.89% | 98.58% | 904 | 89.05% | 98.84% |
| *Mouse* | 475 | 28.57% | 80.06% | 939 | 70.39% | 84.55% |
| *Rat* | 444 | 24.82% | 79.92% | 890 | 62.36% | 84.81% |
| *Dog* | 447 | 44.04% | 80.47% | 894 | 70.47% | 86.81% |
| *Opossum* | 426 | 7.43% | 85.11% | 830 | 38.92% | 86.52% |
| *Chicken* | 320 | 4.60% | 87.85% | 614 | 25.24% | 85.20% |
| *Frog* | 330 | 2.85% | 91.96% | 653 | 5.21% | 86.67% |
| *Zebrafish* | 304 | 3.51% | 91.38% | 606 | 4.95% | 91.12% |

**Supplementary Table S1.B.** Conservation rates of 5Kbp upstream regions and TFBSs as found by the UCSC multiple alignments-based analysis. This table reproduces the information in Table 1 from the main text.

| **Human genes vs.** | ***No. orthol. Genes*** | ***Block Coverage*** | ***Avg. Block Identity*** | ***Detectable Sites*** | ***TFBSs Detected*** | ***Avg. Conserved Site Id.*** |
| --- | --- | --- | --- | --- | --- | --- |
| *Chimp* | 512 | 94.06% | 98.27% | 1157 | 94.81% | 98.74% |
| *Mouse* | 506 | 24.20% | 73.39% | 1146 | 72.34% | 82.91% |
| *Rat* | 496 | 23.09% | 73.21% | 1129 | 67.14% | 83.00% |
| *Dog* | 507 | 46.05% | 75.37% | 1151 | 73.59% | 84.77% |
| *Opossum* | 389 | 6.72% | 74.63% | 912 | 41.23% | 83.93% |
| *Chicken* | 189 | 3.21% | 74.43% | 451 | 21.73% | 85.06% |
| *Frog* | 159 | 3.86% | 73.74% | 395 | 17.97% | 81.42% |
| *Zebrafish* | 125 | 3.62% | 74.05% | 278 | 13.31% | 87.22% |

**Supplementary Table S2:** TFBS conservation dependency on TF identity for human-chimp comparisons. Factors with greater than 7 sites detectable between the two species are shown. Factors are ordered according to *p*-value as calculated by Fisher’s exact test. This table is based on the UCSC multiple alignment analysis methodology presented in the main text.

| **Chimp-Human** | | | |
| --- | --- | --- | --- |
| ***Factor*** | ***Detectable*** | ***% Conserved*** | ***p-value from Fisher exact test*** |
| Sp1 | 114 | 88.60% | 0.002647 |
| HIF-1 | 11 | 72.73% | 0.014605 |
| E2F-1 | 12 | 83.33% | 0.104136 |
| Egr-1 | 12 | 83.33% | 0.104136 |
| POU1F1a | 12 | 83.33% | 0.104136 |
| NF-kappaB | 15 | 86.67% | 0.141729 |
| AhR | 7 | 85.71% | 0.264914 |
| C/EBPalpha | 24 | 100.00% | 0.274924 |
| AP-2alphaA | 23 | 100.00% | 0.290282 |
| AP-1 | 34 | 97.06% | 0.305231 |
| IPF1 | 9 | 88.89% | 0.306537 |
| Gfi1 | 20 | 100.00% | 0.341601 |
| p53 | 22 | 95.45% | 0.375984 |
| CREB | 16 | 100.00% | 0.424117 |
| GATA-1 | 14 | 100.00% | 0.472435 |
| c-Myb | 11 | 100.00% | 0.555226 |
| E2F | 11 | 100.00% | 0.555226 |
| ER-alpha | 11 | 100.00% | 0.555226 |
| HNF-1alpha-A | 11 | 100.00% | 0.555226 |
| MITF | 11 | 100.00% | 0.555226 |
| NF-AT1 | 10 | 100.00% | 0.585873 |
| ATF-2 | 9 | 100.00% | 0.618182 |
| USF1 | 9 | 100.00% | 0.618182 |
| EBF | 8 | 100.00% | 0.652242 |
| IRF-1 | 8 | 100.00% | 0.652242 |
| p50 | 8 | 100.00% | 0.652242 |
| Crx | 7 | 100.00% | 0.688145 |
| GR | 7 | 100.00% | 0.688145 |
| HMG | 7 | 100.00% | 0.688145 |
| HNF-1alpha | 7 | 100.00% | 0.688145 |
| TCF-4 | 7 | 100.00% | 0.688145 |

**Supplementary Table S3:** TFBS conservation dependency on TF identity for human-mouse comparisons. Factors with greater than 7 sites detectable between the two species are shown. Factors are ordered according to *p*-value as calculated by Fisher’s exact test. This table is based on the UCSC multiple alignment analysis methodology presented in the main text.

| **Mouse-Human** | | | |
| --- | --- | --- | --- |
| ***Factor*** | ***Detectable*** | ***% Conserved*** | ***p-value from Fisher exact test*** |
| Gfi1* | 17 | 35.29% | 0.001194 |
| AR | 7 | 14.29% | 0.002203 |
| AP-2alphaA | 23 | 47.83% | 0.007288 |
| Sp1 | 115 | 66.09% | 0.025007 |
| CREB | 17 | 94.12% | 0.02575 |
| ER-alpha | 11 | 45.45% | 0.040525 |
| AP-1 | 34 | 82.35% | 0.068561 |
| Crx | 7 | 42.86% | 0.077237 |
| GATA-1 | 14 | 57.14% | 0.100694 |
| HMG | 7 | 100.00% | 0.102928 |
| EBF | 8 | 50.00% | 0.112024 |
| c-Myb | 11 | 90.91% | 0.118634 |
| NF-kappaB | 14 | 85.71% | 0.142476 |
| NF-AT1 | 10 | 90.00% | 0.14941 |
| C/EBPalpha | 22 | 77.27% | 0.174495 |
| IPF1 | 9 | 88.89% | 0.186226 |
| p53 | 22 | 72.73% | 0.189733 |
| E2F-1 | 12 | 83.33% | 0.198176 |
| HNF-1alpha-A | 11 | 63.64% | 0.201015 |
| TCF-4 | 7 | 57.14% | 0.203178 |
| Egr-1 | 12 | 66.67% | 0.218371 |
| POU1F1a | 12 | 66.67% | 0.218371 |
| HIF-1 | 11 | 81.82% | 0.228587 |
| MITF | 11 | 81.82% | 0.228587 |
| p50 | 8 | 87.50% | 0.22917 |
| E2F | 11 | 72.73% | 0.263112 |
| AhR | 7 | 85.71% | 0.277517 |
| GR | 7 | 85.71% | 0.277517 |
| ATF-2 | 9 | 77.78% | 0.286362 |
| USF1 | 9 | 77.78% | 0.286362 |
| c-Ets-1 | 7 | 71.43% | 0.31928 |
| HNF-1alpha | 7 | 71.43% | 0.31928 |

**Supplementary Table S4:** TFBS conservation dependency on TF identity for human-rat comparisons. Factors with greater than 7 sites detectable between the two species are shown. Factors are ordered according to *p*-value as calculated by Fisher’s exact test. This table is based on the UCSC multiple alignment analysis methodology presented in the main text.

| **Rat-Human** | | | |
| --- | --- | --- | --- |
| ***Factor*** | ***Detectable*** | ***% Conserved*** | ***p-value from Fisher exact test*** |
| Gfi1* | 17 | 29.41% | 0.001246 |
| AR | 7 | 14.29% | 0.005789 |
| TCF-4 | 7 | 14.29% | 0.005789 |
| CREB | 17 | 94.12% | 0.009164 |
| AP-2alphaA | 23 | 43.48% | 0.010587 |
| E2F | 10 | 30.00% | 0.014716 |
| Sp1 | 111 | 60.36% | 0.023498 |
| AP-1 | 33 | 81.82% | 0.028624 |
| GATA-1 | 14 | 42.86% | 0.036845 |
| IRF-1 | 8 | 100.00% | 0.040785 |
| GR | 7 | 100.00% | 0.060933 |
| HMG | 7 | 100.00% | 0.060933 |
| c-Myb | 11 | 90.91% | 0.066551 |
| ER-alpha | 11 | 45.45% | 0.078965 |
| ATF-2 | 9 | 88.89% | 0.121482 |
| p53 | 20 | 60.00% | 0.144516 |
| IPF1 | 8 | 87.50% | 0.161185 |
| p50 | 8 | 87.50% | 0.161185 |
| HNF-1alpha-A | 11 | 54.55% | 0.16246 |
| HIF-1 | 11 | 81.82% | 0.164379 |
| EBF | 8 | 50.00% | 0.165968 |
| C/EBPalpha | 23 | 69.57% | 0.174513 |
| E2F-1 | 12 | 75.00% | 0.217016 |
| NF-kappaB | 14 | 71.43% | 0.218331 |
| NF-AT1 | 10 | 60.00% | 0.225159 |
| Egr-1 | 12 | 66.67% | 0.239586 |
| POU1F1a | 12 | 66.67% | 0.239586 |
| MITF | 11 | 72.73% | 0.242624 |
| Crx | 7 | 57.14% | 0.253011 |
| USF1 | 9 | 66.67% | 0.274106 |
| AhR | 7 | 71.43% | 0.310196 |

**Supplementary Table S5:** TFBS conservation dependency on TF identity for human-dog comparisons. Factors with greater than 7 sites detectable between the two species are shown. Factors are ordered according to *p*-value as calculated by Fisher’s exact test. This table is based on the UCSC multiple alignment analysis methodology presented in the main text.

| **Dog-Human** | | | |
| --- | --- | --- | --- |
| ***Factor*** | ***Detectable*** | ***% Conserved*** | ***p-value from Fisher exact test*** |
| Sp1* | 113 | 54.87% | 2.87E-06 |
| AP-2alphaA* | 23 | 43.48% | 0.001479 |
| Gfi1 | 20 | 45.00% | 0.004369 |
| USF1 | 9 | 33.33% | 0.011118 |
| POU1F1a | 12 | 100.00% | 0.024699 |
| c-Myb | 11 | 100.00% | 0.033681 |
| AP-1 | 34 | 85.29% | 0.047833 |
| NF-kappaB | 15 | 53.33% | 0.049065 |
| p53 | 22 | 59.09% | 0.05715 |
| C/EBPalpha | 23 | 86.96% | 0.069725 |
| IRF-1 | 8 | 100.00% | 0.085242 |
| p50 | 8 | 50.00% | 0.099638 |
| GR | 7 | 100.00% | 0.116091 |
| HNF-1alpha | 7 | 100.00% | 0.116091 |
| E2F-1 | 12 | 58.33% | 0.118907 |
| Egr-1 | 12 | 58.33% | 0.118907 |
| E2F | 11 | 90.91% | 0.134564 |
| HIF-1 | 11 | 90.91% | 0.134564 |
| NF-AT1 | 10 | 90.00% | 0.166562 |
| CREB | 17 | 70.59% | 0.202045 |
| HNF-1alpha-A | 9 | 88.89% | 0.204044 |
| IPF1 | 9 | 88.89% | 0.204044 |
| GATA-1 | 12 | 83.33% | 0.214593 |
| EBF | 8 | 62.50% | 0.223243 |
| MITF | 11 | 81.82% | 0.243274 |
| ER-alpha | 11 | 72.73% | 0.262701 |
| AR | 7 | 85.71% | 0.293748 |
| c-Ets-1 | 7 | 85.71% | 0.293748 |
| Crx | 7 | 85.71% | 0.293748 |
| ATF-2 | 9 | 77.78% | 0.294406 |
| AhR | 7 | 71.43% | 0.317123 |
| HMG | 7 | 71.43% | 0.317123 |
| TCF-4 | 7 | 71.43% | 0.317123 |

**Supplementary Table S6:** TFBS conservation dependency on TF identity for human-opossum comparisons. Factors with greater than 7 sites detectable between the two species are shown. Factors are ordered according to *p*-value as calculated by Fisher’s exact test. This table is based on the UCSC multiple alignment analysis methodology presented in the main text.

| **Opossum-Human** | | | |
| --- | --- | --- | --- |
| ***Factor*** | ***Detectable*** | ***% Conserved*** | ***p-value from Fisher exact test*** |
| HMG | 7 | 100.00% | 0.001959 |
| Gfi1 | 11 | 0.00% | 0.002769 |
| Sp1 | 86 | 29.07% | 0.004883 |
| AhR | 7 | 0.00% | 0.02383 |
| TCF-4 | 7 | 0.00% | 0.02383 |
| CREB | 13 | 69.23% | 0.028725 |
| p50 | 8 | 75.00% | 0.046968 |
| MITF | 10 | 70.00% | 0.048721 |
| ER-alpha | 9 | 11.11% | 0.052143 |
| GATA-1 | 9 | 11.11% | 0.052143 |
| AP-2alphaA | 23 | 26.09% | 0.058059 |
| c-Myb | 11 | 18.18% | 0.077462 |
| C/EBPalpha | 16 | 25.00% | 0.088621 |
| AP-1 | 24 | 50.00% | 0.111193 |
| E2F-1 | 10 | 60.00% | 0.122823 |
| E2F | 11 | 54.55% | 0.15937 |
| POU1F1a | 9 | 55.56% | 0.179367 |
| p53 | 16 | 37.50% | 0.194878 |
| Egr-1 | 8 | 25.00% | 0.196147 |
| GR | 7 | 57.14% | 0.205583 |
| HNF-1alpha | 7 | 57.14% | 0.205583 |
| NF-kappaB | 11 | 36.36% | 0.23213 |
| ATF-2 | 8 | 50.00% | 0.242157 |
| USF1 | 8 | 50.00% | 0.242157 |
| IPF1 | 9 | 44.44% | 0.256515 |
| HNF-1alpha-A | 8 | 37.50% | 0.276305 |
| HIF-1 | 7 | 42.86% | 0.293769 |

**Supplementary Table S7:** TFBS conservation dependency on TF identity for human-chicken comparisons. Factors with greater than 7 sites detectable between the two species are shown. Factors are ordered according to *p*-value as calculated by Fisher’s exact test. This table is based on the UCSC multiple alignment analysis methodology presented in the main text.

| **Chicken-Human** | | | |
| --- | --- | --- | --- |
| ***Factor*** | ***Detectable*** | ***% Conserved*** | ***p-value from Fisher exact test*** |
| CREB | 10 | 60.00% | 0.007716 |
| Sp1 | 47 | 8.51% | 0.008271 |
| c-Myb | 9 | 0.00% | 0.1078 |
| p53 | 8 | 0.00% | 0.138422 |
| IPF1 | 8 | 37.50% | 0.169308 |
| AP-1 | 13 | 30.77% | 0.177476 |
| AhR | 7 | 0.00% | 0.177628 |
| HNF-1alpha | 7 | 0.00% | 0.177628 |
| MITF | 9 | 33.33% | 0.1995 |
| POU1F1a | 9 | 33.33% | 0.1995 |

**Supplementary Table S8:** TFBS conservation dependency on TF identity for human-fugu comparisons. Factors with greater than 7 sites detectable between the two species are shown. Factors are ordered according to *p*-value as calculated by Fisher’s exact test. This table is based on the UCSC multiple alignment analysis methodology presented in the main text.

| **Fugu-Human** | | | |
| --- | --- | --- | --- |
| ***Factor*** | ***Detectable*** | ***% Conserved*** | ***p-value from Fisher exact test*** |
| E2F | 7 | 57.14% | 0.002626 |
| Sp1 | 33 | 0.00% | 0.020398 |
| c-Myb | 8 | 0.00% | 0.4073 |
| AP-1 | 7 | 0.00% | 0.456373 |

**Supplementary Table S9:** TFBS conservation dependency on TF identity for human-tetraodon comparisons. Factors with greater than 7 sites detectable between the two species are shown. Factors are ordered according to *p*-value as calculated by Fisher’s exact test. This table is based on the UCSC multiple alignment analysis methodology presented in the main text.

| **Tetraodon-Human** | | | |
| --- | --- | --- | --- |
| ***Factor*** | ***Detectable*** | ***% Conserved*** | ***p-value from Fisher exact test*** |
| E2F | 9 | 44.44% | 0.013269 |
| Sp1 | 44 | 2.27% | 0.01607 |
| E2F-1 | 8 | 25.00% | 0.190881 |
| c-Myb | 8 | 0.00% | 0.35187 |
| AP-1 | 9 | 11.11% | 0.392509 |

**Supplementary Table S10:** TFBS conservation in relation to the GO category of the related gene (in human-chimp comparisons). The top 30 GO categories in terms of gene numbers in the dataset are shown. GO categories are ordered according to *p*-value as calculated by Fisher’s exact test. This table is based on the UCSC multiple alignment analysis methodology presented in the main text.

| ***Human vs. Chimp*** | | | | | | |
| --- | --- | --- | --- | --- | --- | --- |
| *GO Category* | *Genes* | *5Kbp Upstream Coverage* | *Detectable TFBSs* | *% Detected* | *p-value* | *Significant Over/Under-Conservation* |
| receptor binding | 66 | 97.72% | 251 | 99.60% | 4.85E-06 | Over* |
| extracellular space | 55 | 96.58% | 243 | 99.18% | 6.70E-05 | Over* |
| physiological process | 155 | 94.55% | 535 | 97.38% | 0.000103 | Over* |
| nucleotide binding | 42 | 93.06% | 137 | 87.59% | 0.000208 | Under* |
| extracellular region | 57 | 95.95% | 218 | 98.62% | 0.001283 | Over* |
| plasma membrane | 56 | 91.70% | 140 | 90.00% | 0.005718 | Under |
| response to stress | 93 | 94.93% | 328 | 97.26% | 0.006382 | Over |
| cell-cell signaling | 46 | 96.14% | 154 | 98.70% | 0.006949 | Over |
| response to biotic stimulus | 84 | 94.98% | 287 | 97.21% | 0.012134 | Over |
| cell cycle | 42 | 93.95% | 187 | 91.98% | 0.024193 | Under |
| protein binding | 143 | 94.32% | 474 | 93.46% | 0.02425 | Under |
| transcription | 67 | 96.81% | 223 | 92.83% | 0.042954 | Under |
| mitochondrion organization and biogenesis | 99 | 94.24% | 263 | 93.16% | 0.047237 | Under |
| protein metabolism | 48 | 92.20% | 143 | 92.31% | 0.053996 |  |
| transport | 41 | 92.15% | 149 | 97.32% | 0.057155 |  |
| signal transduction | 117 | 95.38% | 401 | 95.76% | 0.065439 |  |
| catalytic activity | 41 | 92.04% | 101 | 92.08% | 0.074347 |  |
| transcription factor activity | 42 | 97.81% | 137 | 92.70% | 0.074875 |  |
| cell proliferation | 54 | 93.75% | 214 | 93.46% | 0.078596 |  |
| development | 56 | 93.76% | 158 | 93.04% | 0.079537 |  |
| nucleus | 92 | 96.12% | 332 | 93.98% | 0.081008 |  |
| cell death | 48 | 95.39% | 187 | 93.58% | 0.095311 |  |
| response to external stimulus | 66 | 93.25% | 213 | 93.90% | 0.103273 |  |
| regulation of biological process | 156 | 95.12% | 565 | 94.69% | 0.103548 |  |
| biological process | 37 | 94.14% | 106 | 97.17% | 0.108044 |  |
| cell | 117 | 93.81% | 348 | 94.54% | 0.109045 |  |
| binding | 91 | 93.70% | 298 | 94.63% | 0.117391 |  |
| transporter activity | 35 | 92.51% | 123 | 95.93% | 0.155176 |  |
| cytoplasm | 46 | 93.52% | 144 | 95.14% | 0.159652 |  |
| receptor activity | 42 | 92.37% | 114 | 94.74% | 0.173776 |  |

**Supplementary Table S11:** TFBS conservation in relation to the GO category of the related gene (in human-mouse comparisons). The top 30 GO categories in terms of gene numbers in the dataset are shown. GO categories are ordered according to *p*-value as calculated by Fisher’s exact test. This table is based on the UCSC multiple alignment analysis methodology presented in the main text.

| **Human vs Mouse** | | | | | | |
| --- | --- | --- | --- | --- | --- | --- |
| *GO Category* | *Genes* | *5Kbp Upstream Coverage* | *Detectable TFBSs* | *% Detected* | *p-value* | *Significant Over/Under-Conservation* |
| physiological process | 154 | 23.59% | 526 | 78.90% | 1.37E-06 | Over* |
| response to stress | 91 | 23.78% | 316 | 79.11% | 0.00035 | Over* |
| response to biotic stimulus | 81 | 22.67% | 273 | 79.49% | 0.000562 | Over* |
| transcription regulator activity | 34 | 37.65% | 128 | 83.59% | 0.000663 | Over* |
| transporter activity | 35 | 25.00% | 123 | 60.98% | 0.001205 | Under* |
| cell-cell signaling | 44 | 26.00% | 141 | 82.27% | 0.001269 | Over* |
| development | 55 | 35.19% | 157 | 81.53% | 0.001411 | Over* |
| response to external stimulus | 65 | 23.49% | 209 | 79.43% | 0.002559 | Over |
| catalytic activity | 40 | 19.68% | 99 | 61.62% | 0.004632 | Under |
| protein binding | 142 | 26.43% | 464 | 75.86% | 0.004751 | Over |
| regulation of biological process | 155 | 29.96% | 562 | 75.27% | 0.004967 | Over |
| cell proliferation | 53 | 29.13% | 209 | 78.47% | 0.005996 | Over |
| mitochondrion organization and biogenesis | 100 | 25.26% | 266 | 77.07% | 0.008929 | Over |
| receptor binding | 65 | 24.36% | 246 | 77.24% | 0.009744 | Over |
| nucleotide binding | 42 | 23.31% | 137 | 79.56% | 0.01044 | Over |
| binding | 90 | 24.17% | 297 | 68.69% | 0.015795 | Under |
| cell | 118 | 21.23% | 351 | 69.23% | 0.016823 | Under |
| transcription | 67 | 35.72% | 223 | 76.68% | 0.018182 | Over |
| extracellular space | 54 | 23.08% | 232 | 75.86% | 0.026959 | Over |
| extracellular region | 56 | 21.66% | 217 | 76.04% | 0.02733 | Over |
| transport | 39 | 24.11% | 146 | 67.81% | 0.032979 | Under |
| receptor activity | 42 | 24.55% | 114 | 77.19% | 0.042946 | Over |
| nucleus | 92 | 31.28% | 332 | 73.49% | 0.050004 |  |
| signal transduction | 116 | 23.96% | 398 | 71.86% | 0.053253 |  |
| cell cycle | 41 | 28.45% | 182 | 70.88% | 0.063393 |  |
| cell death | 48 | 21.97% | 189 | 73.02% | 0.069469 |  |
| plasma membrane | 57 | 20.12% | 143 | 74.13% | 0.070981 |  |
| cytoplasm | 45 | 22.87% | 136 | 74.26% | 0.071712 |  |
| transcription factor activity | 42 | 36.92% | 137 | 73.72% | 0.076197 |  |
| protein metabolism | 49 | 19.65% | 147 | 72.79% | 0.07827 |  |
| biological process | 35 | 21.69% | 100 | 72.00% | 0.092436 |  |

**Supplementary Table S12:** TFBS conservation in relation to the GO category of the related gene (in human-rat comparisons). The top 30 GO categories in terms of gene numbers in the dataset are shown. GO categories are ordered according to *p*-value as calculated by Fisher’s exact test. This table is based on the UCSC multiple alignment analysis methodology presented in the main text.

| **Human vs. Rat** | | | | | | |
| --- | --- | --- | --- | --- | --- | --- |
| *GO Category* | *Genes* | *5Kbp Upstream Coverage* | *Detectable TFBSs* | *% Detected* | *p-value* | *Significant Over/Under-Conservation* |
| transporter activity | 34 | 22.72% | 117 | 43.59% | 1.71E-08 | Under* |
| cell-cell signaling | 44 | 24.78% | 145 | 84.14% | 4.96E-07 | Over* |
| physiological process | 151 | 21.63% | 521 | 73.32% | 1.11E-05 | Over* |
| receptor binding | 65 | 23.56% | 246 | 76.83% | 6.06E-05 | Over* |
| transport | 39 | 22.59% | 142 | 53.52% | 0.000107 | Under* |
| response to stress | 90 | 21.81% | 319 | 74.29% | 0.000295 | Over* |
| protein complex | 34 | 20.79% | 106 | 52.83% | 0.00047 | Under* |
| response to biotic stimulus | 80 | 20.18% | 276 | 74.64% | 0.000511 | Over* |
| cell proliferation | 53 | 28.20% | 209 | 76.08% | 0.000552 | Over* |
| binding | 90 | 23.16% | 297 | 60.27% | 0.000819 | Under* |
| development | 53 | 31.78% | 151 | 75.50% | 0.004485 | Over |
| protein metabolism | 47 | 19.00% | 140 | 58.57% | 0.005613 | Under |
| cell death | 46 | 21.34% | 187 | 73.26% | 0.010051 | Over |
| regulation of biological process | 151 | 28.06% | 554 | 69.68% | 0.010375 | Over |
| cytoplasm | 45 | 21.91% | 139 | 74.10% | 0.013438 | Over |
| response to external stimulus | 64 | 22.11% | 205 | 72.20% | 0.015421 | Over |
| nucleotide binding | 40 | 23.27% | 131 | 74.05% | 0.015916 | Over |
| extracellular space | 54 | 20.57% | 234 | 71.37% | 0.018992 | Over |
| catalytic activity | 40 | 18.85% | 100 | 60.00% | 0.024975 | Under |
| cell cycle | 41 | 26.57% | 182 | 62.64% | 0.025195 | Under |
| nucleus | 90 | 29.20% | 326 | 65.34% | 0.039557 | Under |
| extracellular region | 55 | 20.26% | 213 | 64.32% | 0.03982 | Under |
| cell | 117 | 20.48% | 349 | 65.90% | 0.04568 | Under |
| signal transduction | 114 | 23.00% | 393 | 66.41% | 0.049211 | Under |
| protein binding | 136 | 25.01% | 456 | 67.11% | 0.051459 |  |
| mitochondrion organization and biogenesis | 97 | 23.67% | 262 | 66.79% | 0.05915 |  |
| transcription | 65 | 32.90% | 217 | 68.20% | 0.060175 |  |
| plasma membrane | 55 | 19.93% | 140 | 67.14% | 0.076533 |  |
| receptor activity | 41 | 24.39% | 113 | 69.03% | 0.076904 |  |
| transcription factor activity | 41 | 34.77% | 132 | 66.67% | 0.077659 |  |

**Supplementary Table S13:** TFBS conservation in relation to the GO category of the related gene (in human-dog comparisons). The top 30 GO categories in terms of gene numbers in the dataset are shown. GO categories are ordered according to *p*-value as calculated by Fisher’s exact test. This table is based on the UCSC multiple alignment analysis methodology presented in the main text.

| **Human vs. Dog** | | | | | | |
| --- | --- | --- | --- | --- | --- | --- |
| *GO Category* | *Genes* | *5Kbp Upstream Coverage* | *Detectable TFBSs* | *% Detected* | *p-value* | *Significant Over/Under-Conservation* |
| cell cycle | 42 | 52.75% | 187 | 56.15% | 6.88E-09 | Under* |
| signal transduction | 118 | 49.88% | 404 | 65.59% | 2.47E-06 | Under* |
| nucleus | 91 | 55.06% | 330 | 66.36% | 0.000137 | Under* |
| nucleotide binding | 42 | 43.44% | 137 | 60.58% | 0.00014 | Under* |
| protein metabolism | 50 | 40.10% | 148 | 84.46% | 0.000323 | Over* |
| protein binding | 144 | 48.31% | 477 | 68.76% | 0.000427 | Under* |
| biological process | 37 | 48.57% | 106 | 61.32% | 0.001259 | Under* |
| cell-cell signaling | 45 | 50.44% | 152 | 82.89% | 0.00133 | Over* |
| response to biotic stimulus | 83 | 47.48% | 284 | 79.58% | 0.001781 | Over |
| regulation of biological process | 156 | 53.94% | 566 | 70.32% | 0.0025 | Under |
| transcription factor activity | 41 | 61.52% | 135 | 64.44% | 0.003429 | Under |
| extracellular region | 56 | 42.96% | 215 | 80.00% | 0.003959 | Over |
| binding | 89 | 47.20% | 291 | 78.69% | 0.004371 | Over |
| cell | 118 | 46.12% | 351 | 69.23% | 0.005019 | Under |
| plasma membrane | 57 | 47.24% | 143 | 65.73% | 0.006417 | Under |
| physiological process | 156 | 47.24% | 537 | 76.16% | 0.009606 | Over |
| response to stress | 93 | 47.64% | 327 | 77.37% | 0.011004 | Over |
| response to external stimulus | 66 | 47.57% | 212 | 78.30% | 0.015632 | Over |
| transcription | 66 | 59.47% | 221 | 69.68% | 0.022976 | Under |
| receptor binding | 66 | 50.80% | 251 | 76.89% | 0.026575 | Over |
| extracellular space | 56 | 50.13% | 245 | 76.33% | 0.036311 | Over |
| cell proliferation | 54 | 56.62% | 214 | 74.77% | 0.062993 |  |
| mitochondrion organization and biogenesis | 99 | 47.64% | 263 | 73.76% | 0.063362 |  |
| cell death | 49 | 47.36% | 190 | 73.68% | 0.07174 |  |
| transcription regulator activity | 34 | 59.52% | 128 | 75.78% | 0.072392 |  |
| transport | 39 | 47.46% | 143 | 74.83% | 0.076622 |  |
| development | 56 | 55.68% | 158 | 73.42% | 0.077096 |  |
| cytoplasm | 45 | 41.98% | 142 | 73.94% | 0.080781 |  |
| catalytic activity | 40 | 41.08% | 99 | 75.76% | 0.085116 |  |
| receptor activity | 42 | 49.68% | 114 | 73.68% | 0.089082 |  |

**Supplementary Table S14:** TFBS conservation in relation to the GO category of the related gene (in human-opossum comparisons). The top 30 GO categories in terms of gene numbers in the dataset are shown. GO categories are ordered according to *p*-value as calculated by Fisher’s exact test. This table is based on the UCSC multiple alignment analysis methodology presented in the main text.

| **Human vs. Opossum** | | | | | | |
| --- | --- | --- | --- | --- | --- | --- |
| *GO Category* | *Genes* | *5Kbp Upstream Coverage* | *Detectable TFBSs* | *% Detected* | *p-value* | *Significant Over/Under-Conservation* |
| physiological process | 122 | 5.63% | 415 | 49.40% | 1.51E-06 | Over* |
| transport | 32 | 5.53% | 102 | 20.59% | 1.85E-06 | Under* |
| binding | 71 | 6.77% | 240 | 29.58% | 5.58E-06 | Under* |
| receptor binding | 51 | 6.49% | 180 | 55.56% | 5.80E-06 | Over* |
| protein metabolism | 41 | 6.27% | 131 | 25.95% | 3.75E-05 | Under* |
| transporter activity | 31 | 6.28% | 91 | 23.08% | 6.74E-05 | Under* |
| protein binding | 122 | 7.01% | 419 | 35.80% | 0.000481 | Under* |
| plasma membrane | 37 | 4.78% | 91 | 28.57% | 0.002996 | Under |
| cell-cell signaling | 35 | 6.37% | 120 | 51.67% | 0.003675 | Over |
| receptor activity | 31 | 6.53% | 88 | 29.55% | 0.005675 | Under |
| nucleotide binding | 32 | 5.82% | 112 | 31.25% | 0.005809 | Under |
| response to external stimulus | 54 | 5.60% | 168 | 48.81% | 0.006116 | Over |
| protein complex | 28 | 5.90% | 84 | 29.76% | 0.007374 | Under |
| response to biotic stimulus | 60 | 5.29% | 209 | 47.37% | 0.007816 | Over |
| DNA binding | 27 | 10.05% | 74 | 29.73% | 0.010786 | Under |
| extracellular space | 43 | 4.08% | 175 | 47.43% | 0.012296 | Over |
| cell cycle | 39 | 7.67% | 176 | 35.23% | 0.013476 | Under |
| signal transduction | 86 | 5.72% | 293 | 44.71% | 0.01951 | Over |
| transcription | 61 | 10.52% | 208 | 45.67% | 0.021298 | Over |
| transcription regulator activity | 32 | 10.15% | 122 | 47.54% | 0.024738 | Over |
| regulation of biological process | 134 | 8.49% | 490 | 43.06% | 0.025857 | Over |
| response to stress | 74 | 6.23% | 268 | 44.03% | 0.031767 | Over |
| cell death | 41 | 6.77% | 164 | 37.80% | 0.043402 | Under |
| transcription factor activity | 40 | 9.80% | 133 | 45.11% | 0.046496 | Over |
| development | 47 | 9.48% | 120 | 45.00% | 0.052501 |  |
| mitochondrion organization and biogenesis | 77 | 6.90% | 213 | 39.44% | 0.052946 |  |
| nucleus | 81 | 10.05% | 305 | 40.66% | 0.055153 |  |
| cell | 82 | 6.11% | 241 | 40.66% | 0.059579 |  |
| cell proliferation | 49 | 7.63% | 196 | 40.31% | 0.062606 |  |
| extracellular region | 44 | 6.17% | 160 | 40.63% | 0.069546 |  |
| cytoplasm | 34 | 6.07% | 97 | 39.18% | 0.079548 |  |

**Supplementary Table S15:** TFBS conservation in relation to the GO category of the related gene (in human-chicken comparisons). The top 30 GO categories in terms of gene numbers in the dataset are shown. GO categories are ordered according to *p*-value as calculated by Fisher’s exact test. This table is based on the UCSC multiple alignment analysis methodology presented in the main text.

| **Human vs. Chicken** | | | | | | |
| --- | --- | --- | --- | --- | --- | --- |
| *GO Category* | *Genes* | *5Kbp Upstream Coverage* | *Detectable TFBSs* | *% Detected* | *p-value* | *Significant Over/Under-Conservation* |
| cell-cell signaling | 22 | 2.53% | 86 | 41.86% | 1.32E-06 | Over* |
| transport | 20 | 3.07% | 68 | 4.41% | 2.31E-05 | Under* |
| protein metabolism | 24 | 4.21% | 82 | 6.10% | 2.52E-05 | Under* |
| response to stress | 28 | 3.92% | 116 | 9.48% | 5.40E-05 | Under* |
| transporter activity | 21 | 3.27% | 61 | 4.92% | 0.000119 | Under* |
| protein binding | 51 | 3.09% | 202 | 14.36% | 0.00024 | Under* |
| protein complex | 16 | 3.20% | 39 | 2.56% | 0.000528 | Under* |
| development | 25 | 3.77% | 67 | 37.31% | 0.00073 | Over* |
| transcription | 32 | 3.73% | 91 | 32.97% | 0.002045 | Over |
| receptor binding | 22 | 2.96% | 84 | 33.33% | 0.002443 | Over |
| response to external stimulus | 20 | 2.67% | 71 | 11.27% | 0.007284 | Under |
| extracellular region | 21 | 3.70% | 87 | 31.03% | 0.008143 | Over |
| transcription factor activity | 22 | 3.53% | 52 | 34.62% | 0.009382 | Over |
| catabolism | 13 | 6.54% | 63 | 11.11% | 0.010474 | Under |
| response to biotic stimulus | 19 | 3.51% | 79 | 12.66% | 0.011051 | Under |
| signal transduction | 35 | 2.17% | 120 | 28.33% | 0.013084 | Over |
| cell | 39 | 2.04% | 126 | 27.78% | 0.015811 | Over |
| cell death | 17 | 4.14% | 80 | 13.75% | 0.019012 | Under |
| cell cycle | 13 | 3.16% | 71 | 14.08% | 0.0298 | Under |
| binding | 37 | 2.85% | 119 | 17.65% | 0.047992 | Under |
| transcription regulator activity | 16 | 5.05% | 63 | 26.98% | 0.06974 |  |
| mitochondrion organization and biogenesis | 29 | 3.22% | 85 | 17.65% | 0.072374 |  |
| biosynthesis | 18 | 3.17% | 47 | 27.66% | 0.08249 |  |
| regulation of biological process | 65 | 3.72% | 242 | 21.49% | 0.090234 |  |
| physiological process | 52 | 2.46% | 201 | 21.89% | 0.091096 |  |
| nucleus | 49 | 4.59% | 177 | 22.03% | 0.092126 |  |
| cytoplasm | 18 | 1.38% | 53 | 16.98% | 0.100182 |  |
| DNA binding | 18 | 4.19% | 45 | 26.67% | 0.100763 |  |
| catalytic activity | 16 | 3.21% | 45 | 22.22% | 0.149028 |  |
| structural molecule activity | 14 | 1.84% | 39 | 20.51% | 0.160347 |  |

**Supplementary Table S16:** TFBS conservation in relation to the GO category of the related gene (in human-fugu comparisons). The top 30 GO categories in terms of gene numbers in the dataset are shown. GO categories are ordered according to *p*-value as calculated by Fisher’s exact test. This table is based on the UCSC multiple alignment analysis methodology presented in the main text.

| **Human vs. Fugu** | | | | | | |
| --- | --- | --- | --- | --- | --- | --- |
| *GO Category* | *Genes* | *5Kbp Upstream Coverage* | *Detectable TFBSs* | *% Detected* | *p-value* | *Significant Over/Under-Conservation* |
| transcription factor activity | 20 | 2.19% | 51 | 39.22% | 6.51E-09 | Over* |
| transcription | 30 | 2.53% | 76 | 28.95% | 5.62E-07 | Over* |
| regulation of biological process | 49 | 3.82% | 167 | 17.37% | 0.000311 | Over* |
| transport | 14 | 3.12% | 56 | 0.00% | 0.000359 | Under* |
| protein metabolism | 13 | 7.58% | 53 | 0.00% | 0.000577 | Under* |
| nucleus | 39 | 4.28% | 127 | 18.90% | 0.000725 | Over* |
| transporter activity | 13 | 3.68% | 47 | 0.00% | 0.001464 | Under* |
| cell cycle | 11 | 4.47% | 56 | 23.21% | 0.003949 | Over |
| extracellular region | 8 | 5.27% | 40 | 0.00% | 0.004206 | Under |
| physiological process | 27 | 2.58% | 95 | 5.26% | 0.006832 | Under |
| cytoplasm | 14 | 1.60% | 34 | 0.00% | 0.010131 | Under |
| response to stress | 18 | 5.66% | 78 | 5.13% | 0.014419 | Under |
| transcription regulator activity | 19 | 2.89% | 60 | 20.00% | 0.018528 | Over |
| mitochondrion organization and biogenesis | 18 | 3.04% | 57 | 19.30% | 0.02981 | Over |
| DNA metabolism | 10 | 4.21% | 25 | 0.00% | 0.036339 | Under |
| response to biotic stimulus | 12 | 3.35% | 47 | 4.26% | 0.04165 | Under |
| cell death | 9 | 8.71% | 57 | 5.26% | 0.042001 | Under |
| response to external stimulus | 10 | 3.23% | 36 | 2.78% | 0.042738 | Under |
| protein complex | 14 | 2.35% | 39 | 20.51% | 0.043885 | Over |
| nucleotide binding | 8 | 5.38% | 38 | 5.26% | 0.096008 |  |
| catalytic activity | 8 | 3.61% | 17 | 0.00% | 0.108774 |  |
| protein binding | 38 | 3.83% | 149 | 11.41% | 0.139889 |  |
| signal transduction | 18 | 2.73% | 57 | 8.77% | 0.140444 |  |
| development | 21 | 2.66% | 48 | 14.58% | 0.14891 |  |
| cell | 13 | 3.14% | 32 | 6.25% | 0.153275 |  |
| cytoskeleton | 11 | 0.57% | 32 | 6.25% | 0.153275 |  |
| DNA binding | 14 | 3.96% | 32 | 6.25% | 0.153275 |  |
| binding | 25 | 3.04% | 76 | 10.53% | 0.153951 |  |
| structural molecule activity | 13 | 0.78% | 41 | 9.76% | 0.196659 |  |
| cell proliferation | 12 | 3.16% | 42 | 11.90% | 0.202278 |  |

**Supplementary Table S17:** TFBS conservation in relation to the GO category of the related gene (in human-tetraodon comparisons). The top 30 GO categories in terms of gene numbers in the dataset are shown. GO categories are ordered according to *p*-value as calculated by Fisher’s exact test. This table is based on the UCSC multiple alignment analysis methodology presented in the main text.

| **Human vs. Tetraodon** | | | | | | |
| --- | --- | --- | --- | --- | --- | --- |
| *GO Category* | *Genes* | *5Kbp Upstream Coverage* | *Detectable TFBSs* | *% Detected* | *p-value* | *Significant Over/Under-Conservation* |
| transcription | 39 | 1.93% | 110 | 28.18% | 4.10E-09 | Over* |
| transcription regulator activity | 21 | 2.95% | 82 | 28.05% | 2.97E-06 | Over* |
| transcription factor activity | 24 | 1.79% | 58 | 29.31% | 6.14E-05 | Over* |
| nucleus | 52 | 2.98% | 178 | 18.54% | 0.000133 | Over* |
| regulation of biological process | 64 | 2.84% | 225 | 16.89% | 0.000134 | Over* |
| transport | 15 | 2.90% | 56 | 0.00% | 0.000373 | Under* |
| protein metabolism | 18 | 5.09% | 64 | 1.56% | 0.001172 | Under* |
| transporter activity | 14 | 3.41% | 46 | 0.00% | 0.001702 | Under |
| DNA metabolism | 13 | 2.77% | 45 | 26.67% | 0.002421 | Over |
| cytoplasm | 15 | 1.43% | 37 | 0.00% | 0.006386 | Under |
| extracellular region | 12 | 4.10% | 50 | 2.00% | 0.007604 | Under |
| response to biotic stimulus | 16 | 2.72% | 67 | 20.90% | 0.010322 | Over |
| cell | 24 | 2.30% | 56 | 3.57% | 0.015537 | Under |
| cell death | 14 | 5.41% | 65 | 4.62% | 0.018801 | Under |
| response to external stimulus | 15 | 2.66% | 59 | 20.34% | 0.020655 | Over |
| nucleotide binding | 13 | 3.36% | 52 | 3.85% | 0.023974 | Under |
| signal transduction | 27 | 2.41% | 76 | 6.58% | 0.040544 | Under |
| protein complex | 15 | 2.13% | 38 | 21.05% | 0.043745 | Over |
| binding | 28 | 2.52% | 80 | 7.50% | 0.058335 |  |
| catalytic activity | 12 | 3.13% | 21 | 0.00% | 0.061017 |  |
| response to stress | 23 | 4.56% | 99 | 15.15% | 0.077342 |  |
| protein binding | 55 | 2.74% | 204 | 13.24% | 0.099412 |  |
| DNA binding | 15 | 3.04% | 37 | 5.41% | 0.100121 |  |
| cell cycle | 20 | 2.47% | 84 | 14.29% | 0.114276 |  |
| physiological process | 39 | 2.12% | 138 | 12.32% | 0.130472 |  |
| plasma membrane | 13 | 2.18% | 33 | 6.06% | 0.137193 |  |
| mitochondrion organization and biogenesis | 29 | 2.02% | 77 | 12.99% | 0.14719 |  |
| structural molecule activity | 15 | 0.72% | 46 | 8.70% | 0.157056 |  |
| development | 22 | 2.20% | 52 | 11.54% | 0.181983 |  |
| cell proliferation | 13 | 2.79% | 42 | 11.90% | 0.198292 |  |

**Supplementary Table S18:** Block coverage rates, TFBS detection rates, and base regulatory potential rates for all 256 combinations of genomes tested. In contrast to the tables in the main text, the rates calculated in this table are calculated in relation to the 513 genes (i.e. 513 x 5Kbp upstream regions = 2,565,000bp) and 1162 TFBSs detectable between human and *any* of the eight other species tested. For each of the 256 combinations, a conserved base or TFBS had to be conserved between human and each of the other listed genomes.

| ***Genome Combination*** | ***% Block coverage (>65% id.)*** | ***Detected Sites*** | ***BRPR*** |
| --- | --- | --- | --- |
| Human-Chimp-Mouse-Opossum-Chicken | 0.94 | 7.75% | 7.628 |
| Human-Chimp-Mouse-Rat-Opossum-Chicken | 0.90 | 7.40% | 7.622 |
| Human-Chimp-Rat-Opossum-Chicken | 0.91 | 7.40% | 7.520 |
| Human-Mouse-Opossum-Chicken | 0.99 | 7.92% | 7.485 |
| Human-Mouse-Rat-Opossum-Chicken | 0.94 | 7.57% | 7.466 |
| Human-Chimp-Opossum-Chicken | 0.96 | 7.75% | 7.455 |
| Human-Rat-Opossum-Chicken | 0.96 | 7.57% | 7.354 |
| Human-Opossum-Chicken | 1.28 | 7.92% | 7.262 |
| Human-Chimp-Mouse-Rat-Chicken | 1.02 | 7.83% | 7.091 |
| Human-Mouse-Rat-Chicken | 1.06 | 8.00% | 7.017 |
| Human-Chimp-Rat-Chicken | 1.04 | 7.83% | 6.950 |
| Human-Mouse-Chicken | 1.13 | 8.35% | 6.939 |
| Human-Chimp-Mouse-Chicken | 1.09 | 8.18% | 6.929 |
| Human-Rat-Chicken | 1.10 | 8.00% | 6.875 |
| Human-Chimp-Mouse-Dog-Opossum-Chicken | 0.90 | 6.28% | 6.654 |
| Human-Chimp-Mouse-Opossum | 4.36 | 30.98% | 6.599 |
| Human-Chimp-Mouse-Rat-Opossum | 4.03 | 28.66% | 6.581 |
| Human-Mouse-Dog-Opossum-Chicken | 0.92 | 6.37% | 6.575 |
| Human-Chicken | 3.21 | 8.43% | 6.556 |
| Human-Chimp-Mouse-Rat-Dog-Opossum-Chicken | 0.86 | 5.94% | 6.534 |
| Human-Chimp-Dog-Opossum-Chicken | 0.92 | 6.28% | 6.487 |
| Human-Chimp-Rat-Dog-Opossum-Chicken | 0.87 | 5.94% | 6.480 |
| Human-Mouse-Rat-Dog-Opossum-Chicken | 0.89 | 6.02% | 6.431 |
| Human-Mouse-Opossum | 4.62 | 31.67% | 6.430 |
| Human-Dog-Opossum-Chicken | 0.94 | 6.37% | 6.415 |
| Human-Mouse-Rat-Opossum | 4.27 | 29.35% | 6.409 |
| Human-Chimp-Rat-Opossum | 4.15 | 28.74% | 6.407 |
| Human-Rat-Dog-Opossum-Chicken | 0.89 | 6.02% | 6.370 |
| Human-Rat-Opossum | 4.40 | 29.43% | 6.247 |
| Human-Chimp-Mouse-Dog-Opossum | 4.01 | 26.59% | 6.240 |
| Human-Chimp-Mouse-Rat-Dog-Chicken | 0.97 | 6.37% | 6.200 |
| Human-Chimp-Mouse-Dog-Chicken | 1.03 | 6.71% | 6.190 |
| Human-Chimp-Mouse-Rat-Dog-Opossum | 3.74 | 24.53% | 6.141 |
| Human-Mouse-Dog-Chicken | 1.04 | 6.80% | 6.137 |
| Human-Mouse-Rat-Dog-Chicken | 0.99 | 6.45% | 6.103 |
| Human-Chimp-Rat-Dog-Chicken | 0.99 | 6.37% | 6.083 |
| Human-Mouse-Dog-Opossum | 4.21 | 27.19% | 6.076 |
| Human-Rat-Dog-Chicken | 1.01 | 6.45% | 6.035 |
| Human-Chimp-Rat-Dog-Opossum | 3.84 | 24.61% | 5.992 |
| Human-Mouse-Rat-Dog-Opossum | 3.94 | 25.13% | 5.971 |
| Human-Dog-Chicken | 1.10 | 6.88% | 5.891 |
| Human-Opossum | 6.73 | 32.36% | 5.876 |
| Human-Chimp-Chicken | 1.30 | 8.26% | 5.864 |
| Human-Chimp-Opossum | 5.02 | 31.67% | 5.845 |
| Human-Rat-Dog-Opossum | 4.04 | 25.22% | 5.843 |
| Human-Chimp-Dog-Chicken | 1.11 | 6.80% | 5.751 |
| Human-Chimp-Dog-Opossum | 4.45 | 26.94% | 5.663 |
| Human-Dog-Opossum | 4.64 | 27.54% | 5.565 |
| Human-Mouse-Dog-Opossum-Tetraodon | 0.63 | 3.70% | 4.876 |
| Human-Dog-Opossum-Tetraodon | 0.63 | 3.70% | 4.833 |
| Human-Mouse-Opossum-Tetraodon | 0.66 | 3.70% | 4.672 |
| Human-Mouse-Rat-Dog-Opossum-Tetraodon | 0.61 | 3.36% | 4.659 |
| Human-Rat-Dog-Opossum-Tetraodon | 0.62 | 3.36% | 4.641 |
| Human-Chimp-Mouse-Dog-Opossum-Tetraodon | 0.62 | 3.44% | 4.634 |
| Human-Opossum-Tetraodon | 0.84 | 3.70% | 4.620 |
| Human-Chimp-Dog-Opossum-Tetraodon | 0.63 | 3.44% | 4.557 |
| Human-Chimp-Mouse-Opossum-Tetraodon | 0.63 | 3.44% | 4.552 |
| Human-Chimp-Opossum-Tetraodon | 0.64 | 3.44% | 4.520 |
| Human-Mouse-Rat-Opossum-Tetraodon | 0.64 | 3.36% | 4.500 |
| Human-Rat-Opossum-Tetraodon | 0.64 | 3.36% | 4.475 |
| Human-Chimp-Mouse-Rat-Dog-Opossum-Tetraodon | 0.60 | 3.10% | 4.443 |
| Human-Chimp-Rat-Dog-Opossum-Tetraodon | 0.61 | 3.10% | 4.419 |
| Human-Chimp-Mouse-Rat-Opossum-Tetraodon | 0.61 | 3.10% | 4.374 |
| Human-Chimp-Rat-Opossum-Tetraodon | 0.62 | 3.10% | 4.342 |
| Human-Mouse-Dog-Tetraodon | 0.75 | 3.79% | 4.265 |
| Human-Chimp-Mouse-Dog-Tetraodon | 0.72 | 3.53% | 4.172 |
| Human-Dog-Tetraodon | 0.77 | 3.79% | 4.165 |
| Human-Mouse-Rat-Dog-Tetraodon | 0.73 | 3.44% | 4.118 |
| Human-Mouse-Tetraodon | 0.79 | 3.79% | 4.103 |
| Human-Rat-Dog-Tetraodon | 0.73 | 3.44% | 4.086 |
| Human-Chimp-Mouse-Rat-Dog-Tetraodon | 0.69 | 3.18% | 4.074 |
| Human-Mouse-Rat-Tetraodon | 0.75 | 3.44% | 4.009 |
| Human-Chimp-Rat-Dog-Tetraodon | 0.71 | 3.18% | 3.986 |
| Human-Chimp-Mouse-Tetraodon | 0.76 | 3.53% | 3.958 |
| Human-Rat-Tetraodon | 0.78 | 3.44% | 3.951 |
| Human-Tetraodon | 2.50 | 3.79% | 3.936 |
| Human-Chimp-Mouse-Rat-Tetraodon | 0.72 | 3.18% | 3.928 |
| Human-Mouse-Dog-Opossum-Fugu-Tetraodon | 0.54 | 2.58% | 3.924 |
| Human-Dog-Opossum-Fugu-Tetraodon | 0.55 | 2.58% | 3.900 |
| Human-Chimp-Dog-Tetraodon | 0.78 | 3.53% | 3.864 |
| Human-Chimp-Rat-Tetraodon | 0.74 | 3.18% | 3.842 |
| Human-Mouse-Dog-Opossum-Fugu | 0.63 | 2.75% | 3.812 |
| Human-Dog-Opossum-Fugu | 0.64 | 2.75% | 3.795 |
| Human-Chimp-Mouse-Dog-Opossum-Fugu-Tetraodon | 0.52 | 2.41% | 3.784 |
| Human-Mouse-Opossum-Fugu-Tetraodon | 0.57 | 2.58% | 3.773 |
| Human-Chimp-Dog-Opossum-Fugu-Tetraodon | 0.52 | 2.41% | 3.765 |
| Human-Opossum-Fugu-Tetraodon | 0.72 | 2.58% | 3.748 |
| Human-Mouse-Rat-Dog-Opossum-Fugu-Tetraodon | 0.53 | 2.24% | 3.653 |
| Human-Rat-Dog-Opossum-Fugu-Tetraodon | 0.53 | 2.24% | 3.647 |
| Human-Chimp-Mouse-Opossum-Fugu-Tetraodon | 0.54 | 2.41% | 3.646 |
| Human-Mouse-Dog-Fugu-Tetraodon | 0.62 | 2.67% | 3.636 |
| Human-Chimp-Opossum-Fugu-Tetraodon | 0.54 | 2.41% | 3.628 |
| Human-Mouse-Opossum-Fugu | 0.67 | 2.75% | 3.622 |
| Human-Dog-Fugu-Tetraodon | 0.63 | 2.67% | 3.616 |
| Human-Opossum-Fugu | 0.87 | 2.75% | 3.604 |
| Human-Chimp-Mouse-Dog-Opossum-Fugu | 0.62 | 2.58% | 3.595 |
| Human-Rat-Dog-Opossum-Fugu | 0.62 | 2.41% | 3.583 |
| Human-Mouse-Rat-Dog-Opossum-Fugu | 0.62 | 2.41% | 3.579 |
| Human-Chimp-Mouse-Dog-Fugu-Tetraodon | 0.60 | 2.50% | 3.547 |
| Human-Chimp-Dog-Opossum-Fugu | 0.63 | 2.58% | 3.544 |
| Human-Mouse-Rat-Opossum-Fugu-Tetraodon | 0.55 | 2.24% | 3.540 |
| Human-Rat-Opossum-Fugu-Tetraodon | 0.55 | 2.24% | 3.533 |
| Human-Mouse-Dog-Fugu | 0.74 | 2.93% | 3.528 |
| Human-Chimp-Mouse-Opossum-Fugu | 0.64 | 2.58% | 3.511 |
| Human-Chimp-Dog-Fugu-Tetraodon | 0.60 | 2.50% | 3.506 |
| Human-Mouse-Fugu-Tetraodon | 0.66 | 2.67% | 3.504 |
| Human-Chimp-Opossum-Fugu | 0.64 | 2.58% | 3.503 |
| Human-Chimp-Mouse-Fugu-Tetraodon | 0.60 | 2.50% | 3.497 |
| Human-Chimp-Fugu-Tetraodon | 0.61 | 2.50% | 3.483 |
| Human-Chimp-Rat-Dog-Opossum-Fugu-Tetraodon | 0.51 | 2.07% | 3.483 |
| Human-Chimp-Mouse-Rat-Dog-Opossum-Fugu-Tetraodon | 0.51 | 2.07% | 3.479 |
| Human-Fugu-Tetraodon | 1.77 | 2.67% | 3.468 |
| Human-Dog-Fugu | 0.76 | 2.93% | 3.457 |
| Human-Rat-Opossum-Fugu | 0.65 | 2.41% | 3.437 |
| Human-Mouse-Rat-Opossum-Fugu | 0.65 | 2.41% | 3.435 |
| Human-Chimp-Mouse-Dog-Fugu | 0.72 | 2.75% | 3.427 |
| Human-Chimp-Mouse-Rat-Dog | 14.13 | 49.40% | 3.424 |
| Human-Mouse-Fugu | 0.78 | 2.93% | 3.409 |
| Human-Rat-Dog-Fugu-Tetraodon | 0.61 | 2.32% | 3.392 |
| Human-Mouse-Rat-Dog-Fugu-Tetraodon | 0.61 | 2.32% | 3.384 |
| Human-Chimp-Mouse-Rat-Dog-Opossum-Fugu | 0.61 | 2.24% | 3.380 |
| Human-Chimp-Rat-Opossum-Fugu-Tetraodon | 0.52 | 2.07% | 3.379 |
| Human-Chimp-Rat-Dog-Opossum-Fugu | 0.61 | 2.24% | 3.372 |
| Human-Chimp-Mouse-Rat-Opossum-Fugu-Tetraodon | 0.52 | 2.07% | 3.372 |
| Human-Mouse-Rat-Dog | 15.05 | 51.38% | 3.362 |
| Human-Mouse-Rat-Dog-Fugu | 0.72 | 2.58% | 3.351 |
| Human-Chimp-Mouse-Rat | 16.85 | 58.61% | 3.340 |
| Human-Rat-Dog-Fugu | 0.73 | 2.58% | 3.329 |
| Human-Chimp-Mouse-Rat-Opossum-Fugu | 0.62 | 2.24% | 3.307 |
| Human-Chimp-Mouse-Rat-Dog-Fugu-Tetraodon | 0.58 | 2.15% | 3.305 |
| Human-Chimp-Rat-Opossum-Fugu | 0.62 | 2.24% | 3.299 |
| Human-Chimp-Rat-Dog-Fugu-Tetraodon | 0.58 | 2.15% | 3.298 |
| Human-Chimp-Mouse-Rat-Dog-Fugu | 0.69 | 2.41% | 3.288 |
| Human-Rat-Fugu-Tetraodon | 0.65 | 2.32% | 3.282 |
| Human-Mouse-Rat-Fugu-Tetraodon | 0.64 | 2.32% | 3.282 |
| Human-Mouse-Rat | 18.10 | 61.10% | 3.277 |
| Human-Chimp-Mouse-Fugu | 0.75 | 2.75% | 3.277 |
| Human-Chimp-Mouse-Rat-Fugu-Tetraodon | 0.59 | 2.15% | 3.266 |
| Human-Mouse-Rat-Fugu | 0.75 | 2.58% | 3.257 |
| Human-Chimp-Rat-Fugu-Tetraodon | 0.59 | 2.15% | 3.251 |
| Human-Chimp-Rat-Dog-Chicken-Fugu-Tetraodon | 0.40 | 1.20% | 3.247 |
| Human-Fugu | 3.24 | 2.93% | 3.243 |
| Human-Chimp-Mouse-Rat-Dog-Chicken-Fugu-Tetraodon | 0.40 | 1.20% | 3.231 |
| Human-Chimp-Mouse-Dog-Chicken-Fugu-Tetraodon | 0.40 | 1.20% | 3.219 |
| Human-Chimp-Rat-Dog-Fugu | 0.70 | 2.41% | 3.215 |
| Human-Chimp-Dog-Chicken-Fugu-Tetraodon | 0.40 | 1.20% | 3.209 |
| Human-Rat-Fugu | 0.78 | 2.58% | 3.207 |
| Human-Chimp-Tetraodon | 0.95 | 3.53% | 3.195 |
| Human-Chimp-Dog-Fugu | 0.77 | 2.75% | 3.178 |
| Human-Chimp-Mouse-Rat-Fugu | 0.71 | 2.41% | 3.169 |
| Human-Mouse-Rat-Dog-Chicken-Fugu-Tetraodon | 0.41 | 1.20% | 3.159 |
| Human-Rat-Dog-Chicken-Fugu-Tetraodon | 0.41 | 1.20% | 3.151 |
| Human-Chimp-Rat-Chicken-Fugu-Tetraodon | 0.41 | 1.20% | 3.145 |
| Human-Chimp-Mouse-Rat-Chicken-Fugu-Tetraodon | 0.41 | 1.20% | 3.127 |
| Human-Mouse-Dog-Chicken-Fugu-Tetraodon | 0.41 | 1.20% | 3.125 |
| Human-Chimp-Mouse-Dog | 18.09 | 57.23% | 3.124 |
| Human-Chimp-Mouse-Rat-Dog-Opossum-Chicken-Fugu-Tetraodon | 0.37 | 1.12% | 3.120 |
| Human-Chimp-Rat-Dog-Opossum-Chicken-Fugu-Tetraodon | 0.37 | 1.12% | 3.118 |
| Human-Dog-Chicken-Fugu-Tetraodon | 0.42 | 1.20% | 3.115 |
| Human-Chimp-Rat-Fugu | 0.73 | 2.41% | 3.105 |
| Human-Chimp-Mouse-Chicken-Fugu-Tetraodon | 0.42 | 1.20% | 3.103 |
| Human-Chimp-Chicken-Fugu-Tetraodon | 0.42 | 1.20% | 3.093 |
| Human-Chimp-Mouse-Dog-Opossum-Chicken-Fugu-Tetraodon | 0.37 | 1.12% | 3.090 |
| Human-Chimp-Dog-Opossum-Chicken-Fugu-Tetraodon | 0.37 | 1.12% | 3.078 |
| Human-Rat-Dog-Chicken-Fugu | 0.47 | 1.29% | 3.078 |
| Human-Chimp-Mouse-Rat-Dog-Chicken-Fugu | 0.47 | 1.29% | 3.076 |
| Human-Chimp-Rat-Dog-Chicken-Fugu | 0.47 | 1.29% | 3.071 |
| Human-Mouse-Dog | 19.17 | 59.29% | 3.070 |
| Human-Mouse-Rat-Dog-Chicken-Fugu | 0.47 | 1.29% | 3.066 |
| Human-Mouse-Rat-Chicken-Fugu-Tetraodon | 0.43 | 1.20% | 3.055 |
| Human-Chimp-Rat-Dog-Opossum-Chicken-Fugu | 0.42 | 1.20% | 3.054 |
| Human-Mouse-Dog-Chicken-Fugu | 0.47 | 1.29% | 3.052 |
| Human-Chimp-Mouse-Rat-Dog-Opossum-Chicken-Fugu | 0.42 | 1.20% | 3.046 |
| Human-Rat-Chicken-Fugu-Tetraodon | 0.43 | 1.20% | 3.045 |
| Human-Dog-Chicken-Fugu | 0.48 | 1.29% | 3.042 |
| Human-Chimp-Mouse-Rat-Chicken-Fugu | 0.47 | 1.29% | 3.030 |
| Human-Chimp-Mouse-Dog-Chicken-Fugu | 0.47 | 1.29% | 3.028 |
| Human-Chimp-Mouse-Dog-Opossum-Chicken-Fugu | 0.43 | 1.20% | 3.028 |
| Human-Mouse-Rat-Dog-Opossum-Chicken-Fugu-Tetraodon | 0.38 | 1.12% | 3.027 |
| Human-Chimp-Rat-Chicken-Fugu | 0.47 | 1.29% | 3.025 |
| Human-Chimp-Dog-Opossum-Chicken-Fugu | 0.43 | 1.20% | 3.023 |
| Human-Rat-Dog-Opossum-Chicken-Fugu-Tetraodon | 0.38 | 1.12% | 3.018 |
| Human-Chimp-Mouse-Rat-Opossum-Chicken-Fugu-Tetraodon | 0.38 | 1.12% | 3.015 |
| Human-Chimp-Rat-Opossum-Chicken-Fugu-Tetraodon | 0.38 | 1.12% | 3.013 |
| Human-Mouse-Chicken-Fugu-Tetraodon | 0.44 | 1.20% | 3.008 |
| Human-Chimp-Chicken-Fugu | 0.48 | 1.29% | 3.000 |
| Human-Chicken-Fugu-Tetraodon | 0.86 | 1.20% | 2.996 |
| Human-Chimp-Dog-Chicken-Fugu | 0.48 | 1.29% | 2.995 |
| Human-Mouse-Dog-Opossum-Chicken-Fugu-Tetraodon | 0.38 | 1.12% | 2.992 |
| Human-Chimp-Rat-Dog | 17.04 | 52.24% | 2.987 |
| Human-Chimp-Mouse-Chicken-Fugu | 0.48 | 1.29% | 2.987 |
| Human-Dog-Opossum-Chicken-Fugu-Tetraodon | 0.39 | 1.12% | 2.981 |
| Human-Mouse-Rat-Dog-Opossum-Chicken-Fugu | 0.44 | 1.20% | 2.971 |
| Human-Rat-Dog-Opossum-Chicken-Fugu | 0.44 | 1.20% | 2.969 |
| Human-Chimp-Mouse-Opossum-Chicken-Fugu-Tetraodon | 0.39 | 1.12% | 2.969 |
| Human-Chimp-Opossum-Chicken-Fugu-Tetraodon | 0.39 | 1.12% | 2.959 |
| Human-Rat-Chicken-Fugu | 0.50 | 1.29% | 2.944 |
| Human-Mouse-Dog-Opossum-Chicken-Fugu | 0.44 | 1.20% | 2.943 |
| Human-Rat-Dog | 18.07 | 54.22% | 2.940 |
| Human-Mouse-Rat-Chicken-Fugu | 0.49 | 1.29% | 2.939 |
| Human-Chimp-Rat-Opossum-Chicken-Fugu | 0.44 | 1.20% | 2.934 |
| Human-Chimp-Mouse-Rat-Dog-Chicken-Tetraodon | 0.44 | 1.20% | 2.934 |
| Human-Dog-Opossum-Chicken-Fugu | 0.44 | 1.20% | 2.932 |
| Human-Mouse-Rat-Dog-Chicken-Tetraodon | 0.44 | 1.20% | 2.928 |
| Human-Chimp-Mouse-Rat-Opossum-Chicken-Fugu | 0.44 | 1.20% | 2.925 |
| Human-Rat-Dog-Chicken-Tetraodon | 0.44 | 1.20% | 2.924 |
| Human-Mouse-Rat-Opossum-Chicken-Fugu-Tetraodon | 0.39 | 1.12% | 2.920 |
| Human-Chimp-Rat-Dog-Chicken-Tetraodon | 0.44 | 1.20% | 2.919 |
| Human-Mouse-Chicken-Fugu | 0.50 | 1.29% | 2.916 |
| Human-Chimp-Mouse-Rat-Chicken-Tetraodon | 0.44 | 1.20% | 2.916 |
| Human-Mouse-Dog-Chicken-Tetraodon | 0.44 | 1.20% | 2.915 |
| Human-Rat-Opossum-Chicken-Fugu-Tetraodon | 0.40 | 1.12% | 2.910 |
| Human-Chimp-Rat-Chicken-Tetraodon | 0.44 | 1.20% | 2.901 |
| Human-Chimp-Mouse-Opossum-Chicken-Fugu | 0.45 | 1.20% | 2.897 |
| Human-Chimp-Mouse | 22.63 | 67.90% | 2.896 |
| Human-Mouse | 24.23 | 71.34% | 2.892 |
| Human-Chimp-Opossum-Chicken-Fugu | 0.45 | 1.20% | 2.892 |
| Human-Chicken-Fugu | 1.14 | 1.29% | 2.891 |
| Human-Dog-Chicken-Tetraodon | 0.45 | 1.20% | 2.888 |
| Human-Chimp-Mouse-Dog-Chicken-Tetraodon | 0.45 | 1.20% | 2.885 |
| Human-Chimp-Mouse-Rat-Dog-Opossum-Chicken-Tetraodon | 0.40 | 1.12% | 2.883 |
| Human-Chimp-Rat-Dog-Opossum-Chicken-Tetraodon | 0.40 | 1.12% | 2.881 |
| Human-Chimp-Chicken-Tetraodon | 0.45 | 1.20% | 2.877 |
| Human-Mouse-Opossum-Chicken-Fugu-Tetraodon | 0.40 | 1.12% | 2.871 |
| Human-Chimp-Mouse-Chicken-Tetraodon | 0.45 | 1.20% | 2.870 |
| Human-Chimp-Mouse-Dog-Opossum-Chicken-Tetraodon | 0.40 | 1.12% | 2.865 |
| Human-Opossum-Chicken-Fugu-Tetraodon | 0.49 | 1.12% | 2.859 |
| Human-Chimp-Dog-Opossum-Chicken-Tetraodon | 0.40 | 1.12% | 2.851 |
| Human-Mouse-Rat-Opossum-Chicken-Fugu | 0.46 | 1.20% | 2.848 |
| Human-Chimp-Dog-Chicken-Tetraodon | 0.45 | 1.20% | 2.844 |
| Human-Rat-Opossum-Chicken-Fugu | 0.46 | 1.20% | 2.843 |
| Human-Mouse-Rat-Chicken-Tetraodon | 0.46 | 1.20% | 2.833 |
| Human-Rat-Chicken-Tetraodon | 0.47 | 1.20% | 2.830 |
| Human-Mouse-Rat-Dog-Opossum-Chicken-Tetraodon | 0.41 | 1.12% | 2.813 |
| Human-Mouse-Chicken-Tetraodon | 0.47 | 1.20% | 2.809 |
| Human-Mouse-Opossum-Chicken-Fugu | 0.46 | 1.20% | 2.808 |
| Human-Opossum-Chicken-Fugu | 0.58 | 1.20% | 2.795 |
| Human-Chimp-Rat | 21.21 | 62.48% | 2.794 |
| Human-Rat-Dog-Opossum-Chicken-Tetraodon | 0.41 | 1.12% | 2.793 |
| Human-Chimp-Rat-Opossum-Chicken-Tetraodon | 0.41 | 1.12% | 2.788 |
| Human-Chimp-Mouse-Rat-Opossum-Chicken-Tetraodon | 0.41 | 1.12% | 2.788 |
| Human-Mouse-Dog-Opossum-Chicken-Tetraodon | 0.41 | 1.12% | 2.784 |
| Human-Chicken-Tetraodon | 0.98 | 1.20% | 2.775 |
| Human-Rat | 23.12 | 65.23% | 2.767 |
| Human-Chimp-Mouse-Opossum-Chicken-Tetraodon | 0.41 | 1.12% | 2.759 |
| Human-Dog-Opossum-Chicken-Tetraodon | 0.42 | 1.12% | 2.757 |
| Human-Chimp-Opossum-Chicken-Tetraodon | 0.42 | 1.12% | 2.746 |
| Human-Mouse-Rat-Opossum-Chicken-Tetraodon | 0.42 | 1.12% | 2.717 |
| Human-Rat-Opossum-Chicken-Tetraodon | 0.43 | 1.12% | 2.697 |
| Human-Mouse-Opossum-Chicken-Tetraodon | 0.43 | 1.12% | 2.676 |
| Human-Opossum-Chicken-Tetraodon | 0.53 | 1.12% | 2.649 |
| Human-Chimp-Fugu | 0.94 | 2.75% | 2.625 |
| Human-Chimp-Dog | 43.41 | 70.65% | 1.561 |
| Human-Dog | 46.11 | 72.89% | 1.539 |
| Human-Chimp | 94.04 | 94.41% | 1.009 |

**Supplementary Table S19: Reanalysis of 5Kbp upstream coverage rates and regulatory site conservation using only those sites/regulated genes stored in TRANSFAC public (v. 7.0).**

|  | ***P r o m o t e r s*** | | | ***S I t e s*** | | | |
| --- | --- | --- | --- | --- | --- | --- | --- |
| **Human *vs.*** | ***No. orthol. genes*** | ***Block coverage*** | ***Avg. Block Identity*** | ***Detectable*** | ***% detected*** | ***Avg. nt site identity*** | ***BRPR*** |
| *Chimp* | 271 | 95.36% | 98.23% | 529 | 94.71% | 98.77% | 0.987499 |
| *Mouse* | 266 | 22.64% | 73.09% | 518 | 75.10% | 81.17% | 3.220897 |
| *Rat* | 258 | 21.69% | 72.98% | 504 | 68.45% | 81.59% | 3.0198 |
| *Dog* | 266 | 43.81% | 75.14% | 523 | 76.48% | 82.94% | 1.665525 |
| *Opossum* | 208 | 6.24% | 74.03% | 422 | 40.28% | 82.75% | 5.799083 |
| *Chicken* | 104 | 3.12% | 74.32% | 235 | 19.15% | 83.53% | 6.350927 |
| *Fugu* | 72 | 3.18% | 72.15% | 162 | 10.49% | 82.73% | 3.256322 |
| *Tetraodon* | 89 | 2.59% | 72.78% | 190 | 15.26% | 77.60% | 5.72823 |

**Supplementary Table S20: List of intergenic miRNA genes used in the analysis.**

| **miRNA name** | **Chrom** | **Orient.** | **Gene begin** |
| --- | --- | --- | --- |
| hsa-mir-200b | chr1 | + | 1092347 |
| hsa-mir-200a | chr1 | + | 1093106 |
| hsa-mir-429 | chr1 | + | 1094248 |
| hsa-mir-34a | chr1 | - | 9134422 |
| hsa-mir-552 | chr1 | - | 34907881 |
| hsa-mir-101-1 | chr1 | - | 65296778 |
| hsa-mir-137 | chr1 | - | 98284314 |
| hsa-mir-197 | chr1 | + | 109943038 |
| hsa-mir-135b | chr1 | - | 203684148 |
| hsa-mir-29c | chr1 | - | 206041906 |
| hsa-mir-29b-2 | chr1 | - | 206042490 |
| hsa-mir-205 | chr1 | + | 207672101 |
| hsa-mir-217 | chr2 | - | 56063714 |
| hsa-mir-216 | chr2 | - | 56069697 |
| hsa-mir-10b | chr2 | + | 176723277 |
| hsa-mir-375 | chr2 | - | 219574673 |
| hsa-mir-563 | chr3 | + | 15890282 |
| hsa-mir-138-1 | chr3 | + | 44130708 |
| hsa-mir-135a-1 | chr3 | - | 52303363 |
| hsa-mir-551b | chr3 | + | 169752336 |
| hsa-mir-572 | chr4 | + | 10979549 |
| hsa-mir-573 | chr4 | - | 24131010 |
| hsa-mir-9-2 | chr5 | - | 87998512 |
| hsa-mir-143 | chr5 | + | 148788674 |
| hsa-mir-145 | chr5 | + | 148790402 |
| hsa-mir-219-1 | chr6 | + | 33283590 |
| hsa-mir-206 | chr6 | + | 52117106 |
| hsa-mir-133b | chr6 | + | 52121680 |
| hsa-mir-30c-2 | chr6 | - | 72143454 |
| hsa-mir-30a | chr6 | - | 72170044 |
| hsa-mir-148a | chr7 | - | 25956130 |
| hsa-mir-196b | chr7 | - | 27175706 |
| hsa-mir-129-1 | chr7 | + | 127635161 |
| hsa-mir-182 | chr7 | - | 129197567 |
| hsa-mir-183 | chr7 | - | 129202089 |
| hsa-mir-29a | chr7 | - | 130212108 |
| hsa-mir-29b-1 | chr7 | - | 130212837 |
| hsa-mir-124a-1 | chr8 | - | 9798391 |
| hsa-mir-383 | chr8 | - | 14755389 |
| hsa-mir-320 | chr8 | - | 22158500 |
| hsa-mir-124a-2 | chr8 | + | 65454260 |
| hsa-mir-30b | chr8 | - | 135882031 |
| hsa-mir-30d | chr8 | - | 135886369 |
| hsa-mir-31 | chr9 | - | 21502183 |
| hsa-let-7a-1 | chr9 | + | 95978060 |
| hsa-let-7f-1 | chr9 | + | 95978450 |
| hsa-let-7d | chr9 | + | 95980937 |
| hsa-mir-147 | chr9 | - | 122047148 |
| hsa-mir-219-2 | chr9 | - | 130194813 |
| hsa-mir-606 | chr10 | + | 76982222 |
| hsa-mir-607 | chr10 | - | 98578510 |
| hsa-mir-146b | chr10 | + | 104186259 |
| hsa-mir-202 | chr10 | - | 134911114 |
| hsa-mir-210 | chr11 | - | 558197 |
| hsa-mir-129-2 | chr11 | + | 43559520 |
| hsa-mir-130a | chr11 | + | 57165247 |
| hsa-mir-194-2 | chr11 | - | 64415486 |
| hsa-mir-34b | chr11 | + | 110888873 |
| hsa-mir-34c | chr11 | + | 110889374 |
| hsa-mir-125b-1 | chr11 | - | 121475761 |
| hsa-let-7a-2 | chr11 | - | 121522510 |
| hsa-mir-100 | chr11 | - | 121528225 |
| hsa-mir-200c | chr12 | + | 6943123 |
| hsa-mir-141 | chr12 | + | 6943521 |
| hsa-mir-196a-2 | chr12 | + | 52671789 |
| hsa-let-7i | chr12 | + | 61283733 |
| hsa-mir-492 | chr12 | + | 93752305 |
| hsa-mir-331 | chr12 | + | 94226327 |
| hsa-mir-135a-2 | chr12 | + | 96481721 |
| hsa-mir-622 | chr13 | + | 89681437 |
| hsa-mir-345 | chr14 | + | 99843949 |
| hsa-mir-493 | chr14 | + | 100405150 |
| hsa-mir-337 | chr14 | + | 100410583 |
| hsa-mir-127 | chr14 | + | 100419069 |
| hsa-mir-432 | chr14 | + | 100420573 |
| hsa-mir-370 | chr14 | + | 100447229 |
| hsa-mir-379 | chr14 | + | 100558156 |
| hsa-mir-299 | chr14 | + | 100559884 |
| hsa-mir-380 | chr14 | + | 100561107 |
| hsa-mir-323 | chr14 | + | 100561822 |
| hsa-mir-329-2 | chr14 | + | 100563190 |
| hsa-mir-494 | chr14 | + | 100565724 |
| hsa-mir-495 | chr14 | + | 100569845 |
| hsa-mir-368 | chr14 | + | 100575780 |
| hsa-mir-376a-2 | chr14 | + | 100576159 |
| hsa-mir-376a-1 | chr14 | + | 100576872 |
| hsa-mir-381 | chr14 | + | 100582010 |
| hsa-mir-382 | chr14 | + | 100590396 |
| hsa-mir-134 | chr14 | + | 100590777 |
| hsa-mir-485 | chr14 | + | 100591509 |
| hsa-mir-453 | chr14 | + | 100592280 |
| hsa-mir-154 | chr14 | + | 100595845 |
| hsa-mir-496 | chr14 | + | 100596663 |
| hsa-mir-377 | chr14 | + | 100598140 |
| hsa-mir-409 | chr14 | + | 100601390 |
| hsa-mir-410 | chr14 | + | 100602002 |
| hsa-mir-656 | chr14 | + | 100602814 |
| hsa-mir-203 | chr14 | + | 103653495 |
| hsa-mir-422a | chr15 | - | 61950270 |
| hsa-mir-184 | chr15 | + | 77289185 |
| hsa-mir-7-2 | chr15 | + | 86956060 |
| hsa-mir-9-3 | chr15 | + | 87712252 |
| hsa-mir-365-1 | chr16 | + | 14310643 |
| hsa-mir-138-2 | chr16 | + | 55449931 |
| hsa-mir-132 | chr17 | - | 1900051 |
| hsa-mir-212 | chr17 | - | 1900423 |
| hsa-mir-195 | chr17 | - | 6861743 |
| hsa-mir-497 | chr17 | - | 6862064 |
| hsa-mir-144 | chr17 | - | 24212761 |
| hsa-mir-193a | chr17 | + | 26911128 |
| hsa-mir-365-2 | chr17 | + | 26926543 |
| hsa-mir-10a | chr17 | - | 44012307 |
| hsa-mir-196a-1 | chr17 | - | 44064919 |
| hsa-mir-142 | chr17 | - | 53763677 |
| hsa-mir-21 | chr17 | + | 55273409 |
| hsa-mir-633 | chr17 | + | 58375308 |
| hsa-mir-187 | chr18 | - | 31738886 |
| hsa-mir-122a | chr18 | + | 54269286 |
| hsa-mir-23a | chr19 | - | 13808472 |
| hsa-mir-181c | chr19 | + | 13846513 |
| hsa-mir-150 | chr19 | - | 54695936 |
| hsa-mir-99b | chr19 | + | 56887677 |
| hsa-mir-125a | chr19 | + | 56888319 |
| hsa-mir-371 | chr19 | + | 58982741 |
| hsa-mir-373 | chr19 | + | 58983771 |
| hsa-mir-663 | chr20 | - | 26136913 |
| hsa-mir-296 | chr20 | - | 56826143 |
| hsa-mir-124a-3 | chr20 | + | 61280297 |
| hsa-mir-155 | chr21 | + | 25868163 |
| hsa-mir-648 | chr22 | - | 16843726 |
| hsa-mir-130b | chr22 | + | 20337593 |
| hsa-mir-658 | chr22 | - | 36570323 |
| hsa-let-7a-3 | chr22 | + | 44887293 |
| hsa-let-7b | chr22 | + | 44888230 |
| hsa-mir-221 | chrX | - | 45490637 |
| hsa-mir-222 | chrX | - | 45491473 |
| hsa-mir-223 | chrX | + | 65155437 |
| hsa-mir-374 | chrX | - | 73423916 |
| hsa-mir-384 | chrX | - | 76056178 |
| hsa-mir-325 | chrX | - | 76142316 |
| hsa-mir-220 | chrX | - | 122523735 |
| hsa-mir-106a | chrX | - | 133131973 |
| hsa-mir-450-2 | chrX | - | 133502302 |
| hsa-mir-424 | chrX | - | 133508406 |
| hsa-mir-505 | chrX | - | 138834055 |
